# SLC6A14-mediated glutamine promotes SYTL4–CXCL8 axis activation to drive gemcitabine resistance and immune evasion in pancreatic cancer

**DOI:** 10.1038/s12276-025-01596-w

**Published:** 2025-12-25

**Authors:** Hyeon Woong Kang, Ju Hyun Kim, Jae Woong Jeong, Sungsoon Fang, Won-Gun Yun, Hye-Sol Jung, Wooil Kwon, Jin-Young Jang, Hyo Jung Kim, Joon Seong Park

**Affiliations:** 1 Brain Korea 21 PLUS Project for Medical Science, Yonsei University, College of Medicine, Seoul, Republic of Korea; 2 Department of Medicine, Yonsei University College of Medicine, Seoul, Republic of Korea; 3 Department of Surgery and Cancer Research Institute, Seoul National University College of Medicine, Seoul, Republic of Korea; 4 Biomedical Research Institute, Seoul National University Hospital, Seoul, Republic of Korea

**Keywords:** Cancer metabolism, Cancer microenvironment, Pancreatic cancer

## Abstract

Chemoresistance remains a major challenge in pancreatic ductal adenocarcinoma (PDAC). Glutamine sustains drug resistance and shapes the immunosuppressive tumor microenvironment; however, the underlying mechanisms remain unclear. Identifying key regulators that drive both gemcitabine resistance and immune evasion is crucial for improving theapeutic outcomes in PDAC. Here we identified solute-carrier family 6 member 14 (SLC6A14) as the central regulator of glutamine metabolism that drives gemcitabine resistance. SLC6A14-mediated glutamine metabolism facilitated α-ketoglutarate production, activating mTOR/NF-κB signaling to upregulate PD-L1 expression, playing a central role in immune evasion. Moreover, SLC6A14 induced CXC motif chemokine ligand 8 secretion via synaptotagmin-like 4-mediated exocytosis, paracrinally activating CXCR2 signaling in cancer-associated fibroblasts to enhance mitochondrial fission and amino acid recycling, supporting PDAC progression. Targeting SLC6A14 with α-methyl-tryptophan enhanced gemcitabine sensitivity, suppressed PD-L1 driven immune evasion and reduced tumor growth, metastasis and glutamine production in vivo. These findings underscore SLC6A14 as a pivtoal mediator of glutamine-driven gemcitabine resistance and immune evasion in PDAC. Therapeutic strategies targeting SLC6A14, either alone or in combination with PD-L1 blockade, hold promise for overcoming chemoresistance and enhancing antitumor immunity in gemcitabine-resistant pancreatic cancer.

## SLC6A14 drives gemcitabine resistance in pancreatic cancer

Pancreatic ductal adenocarcinoma (PDAC) is a deadly cancer with low survival rates, and resistance to the drug gemcitabine is a major treatment challenge. Researchers aimed to understand how the tumor environment contributes to this resistance. The study focused on a protein called SLC6A14, which helps transport amino acids into cells. This protein is linked to cancer progression and drug resistance. Researchers used various methods, including cell cultures and animal models, to study the role of SLC6A14 in PDAC. They found that SLC6A14 increases the uptake of glutamine, which fuels cancer cell growth and helps them evade the immune system. By blocking SLC6A14, they reduced cancer cell growth and improved immune response against the tumor. The study concludes that targeting SLC6A14 could be a promising strategy to overcome drug resistance in PDAC.

This summary was initially drafted using artificial intelligence, then revised and fact-checked by the author.

## Introduction

Pancreatic ductal adenocarcinoma (PDAC) is one of the most lethal cancers, with a 5-year survival rate of 12%, and overcoming gemcitabine resistance remains a major challenge in treatment^1–4^. The tumor microenvironment (TME) contributes notably to gemcitabine resistance by providing oncogenic factors that drive PDAC progression^5–8^. The abnormal environment is composed of heterogeneous noncancerous cells, including fibroblasts, immune cells, signaling factors and the extracellular matrix^9–11^. Among the components of the TME, fibroblasts, which are found in both primary and metastatic tumor sites, are phenotypically distinct cells that play crucial roles in tissue generation and wound recovery^12^. Tumor tissues release juxtacrine or paracrine factors that transform nearby fibroblasts into cancer-associated fibroblasts (CAFs) and vice versa^13,14^. CAFs provide various cytokines, chemo-attractants and growth ingredients to initiate malignancy, including cellular proliferation in primary tumors and metastasis to distant areas^15^. However, metabolic interactions between pancreatic cancer cells and CAFs in gemcitabine-resistant environments remain poorly understood.

The solute carrier (SLC) superfamily comprises membrane-associated transporters responsible for the translocation of diverse substrates, including non-essential amino acids, across cellular membranes^16^. With approximately 450 members categorized into 66 families, SLC transporters are integral to metabolic reprogramming, which is a hallmark of cancer progression^17–19^. Among these transporter families, SLC family 6 member 14 (SLC6A14), also known as ATB^0,+^, functions as a Na^+^/Cl^−^-coupled symporter for neutral and cationic amino acids. SLC6A14 has been implicated in oncogenesis owing to its role in regulating extracellular glutamine influx, a critical driver of cancer progression^20,21^.

Aberrant SLC6A14 expression has been reported in multiple cancer types, where it promotes metabolic and signaling alterations. In colorectal cancer, SLC6A14 enhances serine synthesis via JAK2/STAT3 signaling^22,23^, while its inhibition suppresses Wnt/β-catenin signaling and reduces tumor growth^24^. In breast cancer, SLC6A14-mediated amino acid uptake activates mTORC1 signaling, which drives tumor progression and metastasis^25^. In cervical cancer, SLC6A14 supports malignancy by mediating arginine transport^26^.

Beyond its metabolic functions, glutamine availability is closely linked to immune evasion, because glutamine depletion and PD-L1 inhibition synergistically suppress triple-negative breast cancer^27^. Moreover, targeting glutamine transporters, such as SLC25A22, enhances immunotherapeutic efficacy in KRAS-mutant colorectal cancer^28^. While the role of PD-L1 in immune evasion during chemoresistance remains to be fully defined, increasing evidence suggests that gemcitabine resistance may involve immune modulation through mechanisms such as PD-L1 expression. Thus, targeting both metabolic pathways and immune evasion could offer a synergistic approach to overcome chemoresistance and improve therapeutic outcomes in PDAC.

In this study, we propose that targeting SLC6A14 could overcome chemoresistance and immune evasion in PDAC. By regulating glutamine influx, SLC6A14 drives metabolic reprogramming and immune modulation in the TME. Mechanistically, SLC6A14 promotes CXC motif chemokine ligand 8 (CXCL8) secretion via mTOR/NF-κB signaling, which activates CAFs through CXCR2, inducing mitochondrial fission and glutamine production. Genomic analysis of gemcitabine-resistant (GR)-PDAC cells identified synaptotagmin-like protein 4 (SYTL4) as a key downstream effector of SLC6A14, which drives CXCL8 secretion and is linked to gemcitabine resistance and poor prognosis. The reciprocal metabolic crosstalk between cancer cells and the stroma sustains tumor progression. Therefore, targeting SLC6A14 or glutamine metabolism may offer a promising therapeutic strategy to overcome gemcitabine resistance by disrupting tumor–stroma interactions and restoring immune surveillance.

## Materials and methods

### Reagents and antibodies

α-Methyl-DL-tryptophan (α-MT; cat. no. M8377) was purchased from Sigma-Aldrich. Glutamine Assay Kit (cat. no. ab197011) was purchased from Abcam. Human IL-8/CXCL8 Quantikine ELISA Kit (cat. no. D8000C) was purchased from R&D Systems. Human SLC6A14 shRNA lentiviral particles (cat. no. TL309295V) were purchased from OriGene Technology.

Antibodies for western blot were as follows: anti-Granuphilin-A (cat. no. sc-374544, 1:1000), anti-NRF2 (cat. no. sc-365949, 1:1000), anti-GPX4 (cat. no. sc-166570, 1:1000), anti-GAPDH (cat. no. sc-365062, 1:1000), anti-OCT3/4 (cat. no. sc-5279, 1:1000), anti-DRP1 (cat. no. sc-101270, 1:1000) and anti-OPA1 (cat. no. sc-393296, 1:1000) antibodies, purchased from Santa Cruz; anti-E-cadherin (cat. no. 610181, 1:1000) and anti-N-cadherin (cat. no. 610920, 1:1000) antibodies, purchased from BD BioScience; anti-EpCAM (cat. no. 2929S, 1:1000), anti-KLF4 (cat. no. 12173S, 1:1000), anti-phospho-mTOR (cat. no. 2974S, 1:1000), anti-mTOR (cat. no. 2983S, 1:1000), anti-p-NF-κB (cat. no. 3033S, 1:1000), anti-NF-κB (cat. no. 8242S, 1:1000), anti-LC3B (cat. no. 2775S, 1:1000) and anti-p-DRP1 (cat. no. 3455S, 1:1000) antibodies, purchased from Cell Signaling Technology; anti-SLC6A14 (cat. no. PA5-87998, 1:1000) antibody, purchased from Thermo Fisher Scientific; anti-RRM1 (cat. no. ab137114, 1:1000), anti-alpha smooth muscle actin (cat. no. ab5694, 1:1000) and anti-Ki-67 (cat. no. ab15580, 1:1000) antibodies, purchased from Abcam; horseradish peroxidase (HRP)-conjugated goat anti-mouse secondary (cat. no. 7076S, 1:1000) and HRP-conjugated goat anti-rabbit secondary (cat. no. 7074S, 1:1000) antibodies, purchased from Cell Signaling Technology.

Antibodies for immunofluorescent staining were as follows: goat anti-rabbit IgG (H + L) cross-adsorbed secondary antibody, Texas Red-X (cat. no. T-6391, 1:50) and goat anti-mouse IgG (H + L) cross-adsorbed secondary antibody, Alex Fluor 488 (cat. no. A-11001, 1:100), purchased from Invitrogen.

### Human tissue samples

Our research complies with the ethical guidelines of the Declaration of Helsinki and was approved by the institutional review board of Gangnam Severance Hospital (no. 3-2021-0414). Biospecimens (tissue) were collected from patients diagnosed with PDAC and patients with normal pancreas undergoing a surgical resection or tissue biopsy at Gangnam Severance Hospital.

### Cell lines

AsPC-1 (RRID: CVCL_0152), BxPC-3 (RRID: CVCL_0186) and CAPAN-1 (RRID: CVCL_0237) cells were cultured in normal Roswell Park Memorial Institute (RPMI) 1640 with L-glutamine and supplemented with 10% fetal bovine serum (FBS) and 1% antibiotic–antimycotic reagent (Gibco, cat. no. 15240-062) at 37 °C with 5% CO_2_. PANC-1 (RRID: CVCL_0480) and MIA PaCa-2 (RRID: CVCL_0428) cells were cultured in normal Dulbecco’s modified Eagle medium (DMEM) with L-glutamine and sodium pyruvate and supplemented with 10% FBS and 1% antibiotic–antimycotic reagent at 37 °C with 5% CO_2_. Gemcitabine-resistant cell lines (CAPAN-1/GR, AsPC-1/GR and BxPC-3/GR) were constructed by exposing to increasing dosages of gemcitabine (YUHAN, cat. no. L01BC05) for 3 months, and then continuously cultured in medium containing 5 μM, 0.1 μM and 1 μM of gemcitabine, respectively.

### Mice

BALB/c male nude mice (obtained from the Model Animal Research Center of Yonsei University, 6 weeks old) were housed five per cage in an animal room maintained at a constant temperature (19–23 °C) and humidity (55% ± 10%), with a 12-h light/dark cycle (lights on at 07:00), and were allowed access to food and water ad libitum. Mice were allowed to acclimatize for 1 week before the experiment and were randomly assigned to experimental groups. All animal studies were conducted in compliance with the National Institutes of Health Guide for the Care and Use of Laboratory Animals and were performed in accordance with protocols approved by the Institutional Animal Care and Use Committee of Seoul Yonsei Pharmaceutical University Experimental Animal Center (approval no. 2022-0061).

### siRNA transfection

CAPAN-1 (4 × 10^5^) cells were seeded in six-well plates and transfected with Silencer *SLC6A14* small interfering RNA (siRNA) (Santa Cruz Biotechnology, cat. no. sc-91007) or Silencer *Granuphilin* siRNA (Santa Cruz Biotechnology, cat. no. sc-45507), purchased from Santa Cruz Biotechnology, using Lipofectamine RNAiMAX (Thermo Fisher Scientific, cat. no. 13778075) in Opti-MEM according to the manufacturer’s instructions. Cells were processed 48–72 h after transfection and collected for further analysis.

### WST assay

AsPC-1 (3 × 10^4^), BxPC-3 (4 × 10^4^) and CAPAN-1 (4 × 10^4^) cells were seeded in 96-well plates in serum-free medium for 24 h. The cells were either treated with α-MT (Sigma-Aldrich, cat. no. M8377) or transfected with indicated siRNA using Lipofectamine RNAiMAX according to the manufacturer’s instructions for 24 h. After 24 h, indicated concentrations of gemcitabine were treated for 24 h. Thereafter, 10% Ez-Cytox reagent (DoGenBio, cat. no. EZ-3000) was replaced by growth medium for the water-soluble tetrazolium salt (WST) assay and incubated at 37 °C for 24 h. The absorbance was measured at 450-nm wavelength using VersaMax microplate reader (Molecular Devices). The IC_50_ value was measured using GraphPad Prism 8.0 software.

### Protein extraction and western blot analysis

The cells were washed with Dulbecco’s phosphate-buffered saline (DPBS), collected and lysed in radioimmunoprecipitation assay buffer comprising 50 mM Tris–HCl, 150 mM NaCl, 1.0% (v/v) NP-40, 0.5% (w/v) sodium deoxycholate, 1.0 mM EDTA, 0.1% (w/v) SDS and 0.01% (w/v) sodium azide at a pH of 7.4 for 30 min at 4 °C. The protein concentration was determined using a Bio-Rad protein assay kit (Bio-Rad). Thirty micrograms of cellular protein mixed with loading buffer were separated by SDS–PAGE, and the fractionated samples were transferred to a polyvinylidene fluoride membrane (Merck Millipore, cat. no. IPVH00010). After blocking nonspecific binding with Tris-buffered saline, 0.1% Tween-20 containing 5% skim milk for 1 h at room temperature, the membranes were immunoblotted with primary antibodies at 4 °C overnight. Subsequently, the membranes were incubated with HRP-conjugated goat anti-rabbit secondary antibody or goat anti-mouse secondary antibody for 1 h at room temperature. The protein bands were exposed to a clarity western enhanced chemiluminescent HRP substrate (Bio-Rad, cat. no. 1705061) and detected on X-ray films or Amersham imager 600 (GE Healthcare Life Sciences). The band intensity was quantified using ImageJ software (National Institutes of Health). Antibodies used in the experiment are listed in Supplementary Table 1.

### Histology and immunohistochemistry

Fresh pancreas, pancreatic tumor and liver tissues from BALB/c nude mice were obtained, perfused with cold phosphate-buffered saline (PBS), fixed in 4% paraformaldehyde, embedded in paraffin to create formalin-fixed paraffin-embedded blocks of tissue, sectioned (5 µm) and stained with hematoxylin and eosin (H&E). For immunohistochemistry staining, the sections were incubated with primary antibodies against SLC6A14, SYTL4, p-mTOR, p-NF-κB, Ki-67 and Cytokeratin 19. The tissue sections were adhered to poly-L-lysine-covered slides and heated for 10 min at 60 °C, incubated with 100% xylene for 20 min and rehydrated with 100%, 95%, 90% and 70% ethanol for 5 min in sequence. The antigens on the tissue sections were unmasked using Target Retrieval Solution Citrate (pH 6.0) (Agilent Dako) for 4 min at 95–99 °C, cooled down for 30 min at room temperature and washed with PBS. The sections were permeabilized with 0.3% Triton X-100 (Sigma-Aldrich), blocked with 10% donkey serum for 1 h and incubated with primary antibodies at 4 °C overnight. Subsequently, the sections were incubated with HRP-conjugated goat anti-rabbit secondary antibody or goat anti-mouse secondary antibody for 2 h at room temperature and mounted with FluoroShield mounting medium with DAPI (Abcam, cat. no. ab104139) for 10 min at room temperature. The immunostained sections were observed under a Carl Zeiss LSM 980 confocal microscope. The antibodies used in this experiment are listed in Supplementary Table 1.

### Glutamine deprivation

For all glutamine-deprivation experiments, cells were cultured in RPMI 1640 without L-glutamine and supplemented with 10% FBS and 1% antibiotic–antimycotic reagent at 37 °C with 5% CO_2_. During nutrient starvation, the medium was replaced every day.

### RNA extraction and quantitative real-time PCR (RT–qPCR)

Total RNA was isolated from collected cells and tissue samples using TRIzol reagent (Sigma-Aldrich, cat. no. 15596-018) according to the manufacturer’s instructions. A total of 1 μg of RNA was used as the template for first-strand cDNA synthesis using the Maxime Reverse Transcription PreMix (Intron, cat. no. 25081) according to the manufacturer’s instructions. RT–qPCR was performed using Power SYBR Green PCR Master Mix (Applied Biosystems, cat. no. 4368706) on the StepOne Real-Time PCR Systems (Applied Biosystems). Primer sequences used in this study are listed in the key resources table. The results were presented as relative mRNA expression level and calculated with the 2^−ΔΔCT^ method using GAPDH as the reference gene. The sequences are listed in Supplementary Table 1.

### ROS measurement

CAPAN-1 (4 × 10^5^) cells were seeded in a six-well plate per well in serum-free medium for 24 h. Cells were either treated with α-MT or transfected with indicated siRNA using Lipofectamine RNAiMAX according to the manufacturer’s instructions or treated with α-MT for 24 h. Thereafter, the cells were incubated in 20 μm 2′7′-dichlorodihydrofluorescein diacetate (DCF-DA; Sigma-Aldrich) for 30 min at 37 °C in the dark. The cells were then washed twice with PBS. The ROS levels were measured using a FACScanto II flow cytometer (BD Bioscience). A minimum of 10,000 events were recorded for each sample.

### Metabolic assays

Oxygen consumption rate (OCR) and extracellular acidification rate (ECAR) measurements were performed using an XF-24 Extracellular Flux Analyzer (Seahorse Bioscience). CAPAN-1 (4 × 10^5^) cells were seeded onto XF-24 plates per well for 24 h and transfected with indicated siRNA using Lipofectamine RNAiMAX according to the manufacturer’s instructions for 24 h. Thereafter, the cells were incubated in XF assay medium for 1 h at 37 °C in a non-CO_2_ incubator and were subjected to stress by sequentially adding 1 µM oligomycin, 2 µM carbonyl cyanide p-(trifluoromethoxy) phenylhydrazone and a 0.5 µM cocktail of rotenone/antimycin A. The measured OCR and ECAR levels were normalized to the total cellular protein concentration.

### Wound healing and invasion assays

For wound scratch assay, CAPAN-1 (8 × 10^4^) cells were plated in serum-free medium and either treated with α-MT or transfected with indicated siRNA or treated with α-MT for 24 h according to the manufacturer’s instructions on a 24-well plate. Thereafter, wound scratches were made, and images of migrating cells were captured at the indicated time points (0, 16 and 24 h) using a microscope. The wound gap was quantified using ImageJ software (RRID: SCR_003070). For invasion assays, 8-µm pore size wells in a Transwell system (Corning, cat. no. 3464) were coated with Matrigel (1:50, Corning) for 1 h at room temperature. CAPAN-1 (8 × 10^4^) cells were plated in serum-free medium for 24 h, treated with α-MT or transfected with indicated siRNA or treated with α-MT according to the manufacturer’s instructions for 24 h, and then seeded on the apical side of the Transwell chamber (24-well insert) in serum-free medium; growth medium was added to the basal compartment. The cells were allowed to invade for 24 h. The invaded cells on the basal side were fixed in 4% paraformaldehyde for 10 min, permeabilized with 100% MeOH for 10 min, stained with 0.2% crystal violet (JUNSEI, cat. no. C1065-25g) and washed multiple times with PBS. The remaining cells on the top of the chamber were gently scraped off using wet cotton swabs. Migrated and invaded cells were quantified via ImageJ software. Migration and invasion assays were performed in triplicate and repeated three times independently.

### Tumor sphere formation assay

CAPAN-1 (6 × 10^3^) cells were resuspended in ultra-low-attachment round-bottom 96-well plates (Corning, cat. no. 7007) in a volume of 200 μl per well in serum-free medium DMEM/F-12 containing 2% B-27 supplement, 20 ng/ml human epidermal growth factor, 10 ng/ml basic human fibroblast growth factor, 1% 2 μg/ml heparin and 1% antibiotic–antimycotic reagent, made fresh once a week. Plates were incubated at 37 °C with 5% CO_2_ in high humidity, and spheroids were maintained by performing 50% medium replenishments twice a week. Tumor spheres from each replicate well (*n* = 8 wells) were measured at 10× magnification.

### RNA sequencing and data analysis

Total RNA was isolated using TRIzol reagent. RNA quality was assessed by Agilent 2100 bioanalyzer using the RNA 6000 Nano Chip (Agilent Technologies), and RNA quantification was performed using ND-2000 Spectrophotometer (Thermo). For control and test RNAs, library construction was performed using the QuantSeq 3′ mRNA-Seq Library Prep kit (Lexogen), according to the manufacturer’s instructions. High-throughput sequencing was performed as 75-bp single-end sequencing using NextSeq 500 (Illumina). QuantSeq 3′ mRNASeq reads were aligned using Bowtie2^29^. Differentially expressed genes were determined on the basis of counts from unique and multiple alignments using coverage in Bedtools^30^. RC (Read Count) data were processed on the basis of quantile normalization method using EdgeR within R^31^ with Bioconductor^32^. Gene classification was based on searches performed using DAVID (http://david.abcc.ncifcrf.gov/). Gene set enrichment analysis (GSEA) of the ranked gene list was performed using the Java implementation of GSEA obtained from http://www.broadinstitute.org/gsea/ (1000 permutations, minimum term size of 15, maximum term size of 500). Differentially expressed genes between tumor and normal, siCon and siSLC6A14 were analyzed (fold <2.0, false discovery rate (FDR) *P* value <0.05). The analysis included gene sets from Molecular Signature Database (MSigDB) pathways (Broad Institute, http://broadinstitute.org), C5: ontology gene sets (c5.all.v2023.2.Hs.symbols.gmt). The normalized enrichment score (NES) accounts for differences in gene set size. The FDR *q* value (the probability that a gene set with a given NES represents a false-positive finding) was used to set a significance threshold.

### Bioinformatics analysis

Bioinformatics analyses were performed on the basis of The Cancer Genome Atlas (TCGA) Pancreatic Cancer (PAAD) cohort using the UCSC Xena Browser. In total, 183 cases were searched to be used for further analysis for the gene expression patterns of *SLC6A14* and *SYTL4* in the primary PDAC tumors. Kaplan–Meier curves for overall survival and disease-specific survival were generated using GraphPad Prism 8.0 software. *SLC6A14* expression data were obtained from PDAC clinical data, GSE183795 and GSE164665, and analyzed using ‘GEO2R: gene expression omnibus 2R’ (https://www.ncbi.nlm.nih.gov/geo/geo2r/). The primary data are available via GEO (http://www.ncbi.nlm.nih.gov/geo/) under series accession numbers. Feature plots for ‘pseudotime’ and ‘*SLC6A14*’ and were visualized as a Uniform Manifold Approximation and Projection (UMAP) plot using the R statistical package with ‘ggplot2’ and ‘seurat’. Clinicopathological features associated with *SLC6A14* expression, measured in transcripts per million, were obtained from the Gene Expression Profiling Interactive Analysis 2 (GEPIA2) portal and the University of Alabama at Birmingham Cancer data Analysis (UALCAN) portal. Correlation analyses of indicated genes were evaluated in the UALCAN portal. The tumor-immune system interactions database (TISIDB) was conformed to investigate the relevance of *ANXA2* or *SYTL4* with *CXCL8*.

### Liquid chromatography–mass spectrometry (LC–MS)

CAPAN-1 (4 × 10^5^) cells were seeded in triplicate wells of six-well plates in serum-free medium. Thereafter, indicated siRNA transfection was conducted using Lipofectamine RNAiMAX according to the manufacturer’s instructions. Then, cells were washed with PBS and incubated with complete or glutamine-free medium for 24 h. Cells were collected, mixed with internal standard (ISTD) solution consisting of sulfadimethoxine in methanol (MeOH), vortexed for 1 min and centrifuged at 18,000*g* for 7 min at 20 °C. The supernatant was collected for LC–MS analysis. For quantitative analysis, 0.1% folic acid in buffer A consisting of distilled water and buffer B consisting of acetonitrile was used for identifying a positive mode, whereas 6.5 mM ammonium bicarbonate in buffer A consisting of distilled water and buffer B consisting of MeOH was used for identifying a negative mode. For qualitative analysis, α-ketoglutarate (α-KG) analytical standard (Merck) was manufactured by concentration with MeOH, mixed with ISTD solution and then measured as standards for LC–MS analysis. Cells were collected, mixed with ISTD solution consisting of sulfadimethoxine in MeOH, vortexed for 1 min and centrifuged for 18,000*g* for 7 min at 20 °C. The supernatant was collected for LC–MS analysis. Intracellular metabolites were measured using an Ultimate 3000 RSLC system (ThermoFisher Scientific) coupled to a Q-Exactive orbitrap plus mass spectrometer (ThemoFisher Scientific). Analytes were separated on a Acquity UPLC BEH C18 (2.1 × 100 mm, 1.7 μm) (ThermoFisher Scientific) using the following elution buffers. A mass range of 80–1000 *m*/*z* was analyzed with polarity switching at a resolution of 70,000. Metabolites were analyzed using Thermo TraceFinder software (Thermo Scientific Fisher).

### Cytokine array

CAPAN-1 (4 × 10^4^) cells were seeded in a six-well plate per well for 24 h in serum-free medium. The cells were transfected with indicated siRNA using Lipofectamine RNAiMAX according to the manufacturer’s instructions for 24 h. Thereafter, cell culture supernatants were removed by centrifugation and assayed using the Proteome Profiler Human XL Cytokine Array Kit (R&D Systems, cat. no. ARY022B) according to the manufacturer’s instructions. The supernatants were incubated for 24 h at 4 °C and then paired with Human XL cytokine detection antibodies and streptavidin-HRP. Each cytokine was developed using chemiluminescence and quantified using ImageJ software (RRID: SCR_003070).

### Enzyme-linked immunosorbent assay (ELISA) assay

CAPAN-1 (4 × 10^4^) cells were seeded in a six-well plate per well for 24 h in serum-free medium. The cells were either treated with α-MT or transfected with indicated siRNA using Lipofectamine RNAiMAX according to the manufacturer’s instructions for 24 h. Thereafter, cell culture supernatants were collected for centrifugation and transferred to a 96-well microplate coated with antibodies specific to human CXCL8 to assess the levels of CXCL8 using Quantikine ELISA kits (R&D Systems, cat. no. S6050). After any nonspecific bound antibodies were washed, polyclonal antibodies conjugated to HRP were added to each well. A color reagent containing hydrogen peroxide and chromogen was then added. Finally, after sufficient binding to the substrate, the color changed to indicate the amount of sample antigen. CXCL8 levels were quantified using VersaMax microplate reader at 450 nm.

### Collection of blood sample and isolation of peripheral blood mononuclear cells

Human blood samples were collected from patients using vacutainer EDTA tubes (BD Biosciences). The whole blood sample was diluted with PBS, gently layered over an equal volume of Ficoll-Paque solution (GE Healthcare, cat. no. 17-1440-03) and centrifuged at 400*g* for 30–40 min. Peripheral blood mononuclear cells were collected from the second layer, and any remaining platelets were washed off using PBS. Cells were resuspended in freezing medium containing 90% FBS and 10% dimethyl sulfoxide and stored at −80 °C.

### T cell isolation and activation

Anti-CD3 (Thermofisher, cat. no. 16-0037-81) was diluted in PBS and precoated in a six-well plate. T cells were isolated using the Pan T cell isolation kit (Miltenyi Biotec, cat. no. 130-096-535) and AutoMACS Pro (Miltenyi Biotec, cat. no. 130-092-545) according to the manufacturer’s instructions. Then, T cells were activated in precoated wells and anti-CD28 (Thermofisher, cat. no. 16-0289-81) was added for 1–3 days.

### T cell-mediated cancer cell killing assay and IFN-γ ELISA

BxPC-3 (1 × 10^4^) and CAPAN-1 (2 × 10^4^) cells were seeded in a 24-well plate and incubated in serum-free medium for 24 h. The cells were then transfected with indicated siRNA using Lipofectamine RNAiMAX, following the manufacturer’s instructions, for 24 h. Subsequently, the transfected cells were cocultured with preactivated T cells for 6 h. After coculture, supernatants were collected, centrifuged and transferred to a 96-well microplate coated with human IFN-γ-specific antibodies. IFN-γ levels were measured using Quantikine ELISA kits (R&D Systems, cat. no. DIF50C) according to the manufacturer’s protocol. Following supernatant removal, cells were washed with PBS, fixed in 4% paraformaldehyde for 30 min and stained with 0.1% crystal violet. Excess staining was removed by repeated PBS washes. The remaining cells were quantified via ImageJ software. All experiments were performed in triplicate.

### Immunoprecipitation assay

After treatment, the cells were washed once with DPBS and lysed for 30 min on ice. The cell lysates were incubated with the indicated antibodies to form antibody–antigen complexes overnight at 4 °C. After incubation, magnetic A/G beads were prewashed with DPBS. The A/G beads were incubated with preformed antigen–antibody complexes for 30 min. The protein A/G beads were washed three times with DPBS, and the complexes were boiled with denaturing elution buffer (2× Laemmli sample buffer, β-mercaptoethanol and DPBS) at 95 °C for 5 min. The immunoprecipitation samples were subjected to SDS–PAGE followed by western blotting using the indicated antibodies. Antibodies used in the experiment are listed in Supplementary Table 1.

### Glutamine assay

CAPAN-1 (4 × 10^4^) cells were seeded in a six-well plate per well for 24 h in serum-free medium. The cells were either treated with α-MT or transfected with indicated siRNA using Lipofectamine RNAiMAX according to the manufacturer’s instructions for 24 h. Thereafter, the supernatant was obtained to yield extracellular glutamine concentration with a glutamine assay kit (Abcam, cat. no. ab197011) according to the manufacturer’s instructions. The absorbance was measured at 450 nm wavelength using VersaMax microplate reader. Tissue samples from BALB/c nude mice were homogenized using Assay buffer XXIX/hydrolysis buffer (10× (v/w), Abcam, Cambridge, UK), centrifuged at 10,000*g* for 10 min at 4 °C. The supernatant was collected for yielding intracellular glutamine concentration with the glutamine assay kit according to the manufacturer’s instructions. The absorbance was measured at 450 nm wavelength using a VersaMax microplate reader.

### Fibroblast isolation

Tissues were collected from patients with PDAC in Gangnam Severance Hospital. The tissues were washed with Hank’s Balanced Salt Solution supplemented with 1% antibiotic–antimycotic reagent, chopped and centrifuged at 1500 rpm for 1 min at room temperature. A total of 1 mg/ml of collagenase P (Sigma-Aldrich, cat. no. 11213865001) was liquefied with Hank’s Balanced Salt Solution supplemented with 1% antibiotic–antimycotic reagent, syringed through a 0.22-µm syringe filter (Merck Millipore) and added into the tissue samples. The samples were collagenized for 30 min at 37 °C, filtered with 70-µm cell strainer (Falcon) and centrifuged at 1200 rpm for 4 min at room temperature. After the supernatant was removed, the pellet was resuspended with RPMI 1640 supplemented with 1% antibiotic–antimycotic reagent and cultured with RPMI 1640 medium in a 100-mm cell culture dish (Nest) for further studies.

### CAF subtype characterization

After initial expansion, fibroblasts were cultured in RPMI 1640 supplemented with 1% antibiotic–antimycotic reagent. For subtype characterization, total RNA was extracted from fibroblasts using TRIzol reagent (Sigma-Aldrich, cat. no. 15596-018) according to the manufacturer’s instructions. RT–qPCR was performed using Power SYBR Green PCR Master Mix (Applied Biosystems, cat. no. 4368706) on the StepOne Real-Time PCR Systems (Applied Biosystems). Gene expression levels of CAF subtype-specific markers were analyzed, including *ACTA2* and *POSTN* (myCAF), *IL6*, *LIF* and *PDGFRA* (iCAF) and *CD74* and *HLA-DRA* (apCAF). Primer sequences are provided in Supplementary Table 1.

### Animal treatment

Male BALB/c nude mice (purchased Orient Bio, 6 weeks old) was allowed to acclimatize for 1 week. For orthotopic xenograft, the pancreas of BALB/c male nude mice was surgically exposed through an abdominal excision under anesthesia via an intraperitoneal injection of Alfaxan (25 mg/kg). CAPAN-1 (1 × 10^6^) cells suspended in PBS with 50% Matrigel (Corning) were directly injected into the pancreas of the mice using a 30 G needle (BD Bioscience). After the operation, the mice were warmed and monitored until conscious and then placed in HEPA-filtered cages with feed and water. After 2 weeks, the mice were randomly separated into a sensitive group (*n* = 5) and two resistance groups (*n* = 5). *SLC6A14* Human shRNA lentiviral particles (4.5 × 10^6^) (Origene, cat. no. TL309295V) were intravenously injected into the retro-orbital venous sinus. After 1 week, gemcitabine (10 mg/kg) was intraperitoneally injected twice a week. After 4 weeks, the pancreas and liver were collected and snap-frozen for further analysis.

For subcutaneous heterotopic xenografts, BALB/c male nude mice were under anesthesia via an intraperitoneal injection of Alfaxan (25 mg/kg). CAPAN-1 (2 × 10^5^) cells suspended in PBS with 50% Matrigel were subcutaneously injected into the flanks of nude mice alone or co-injected with either 3 × 10^5^ normal fibroblast (NF) cells or CAF cells using a 30 G needle. After the operation, the mice were warmed and monitored until conscious and then placed in HEPA-filtered cages with feed and water. After 2 weeks, *SLC6A14* human shRNA lentiviral particles were intravenously injected into the retro-orbital venous sinus. After 1 week, gemcitabine (10 mg/kg) was intraperitoneally injected twice a week. Tumor sizes were measured twice a week using a digital caliper. Tumor volume was calculated according to the following formula: 1/2 × length (mm) × width (mm)^2^. After 6 weeks, mice were euthanized when the largest tumors reached 1200–1400 mm^3^. Pancreas and liver tissues were collected immediately and snap-frozen for further analysis.

### Statistical analysis

Statistical details of experiments can be found in the figure legends. All data are expressed as mean ± s.d. derived from multiple independent experiments. All the statistical analyses were carried out to determine statistical significance using one-way or two-way analysis of variance (ANOVA) in GraphPad Prism 8.0 software (RRID: SCR_002798). RNA-seq data were analyzed via ExDEGA GraphicPlus (Ebiogen). *P* values below 0.05 were considered statistically significant. Statistical significance was indicated as follows: ^#^*P* < 0.05, ^##^*P* < 0.01, ^###^*P* < 0.001; n.s., no significance.

## Results

### Increased expression of SLC6A14 in gemcitabine-resistant PDAC

**Fig. 1:**
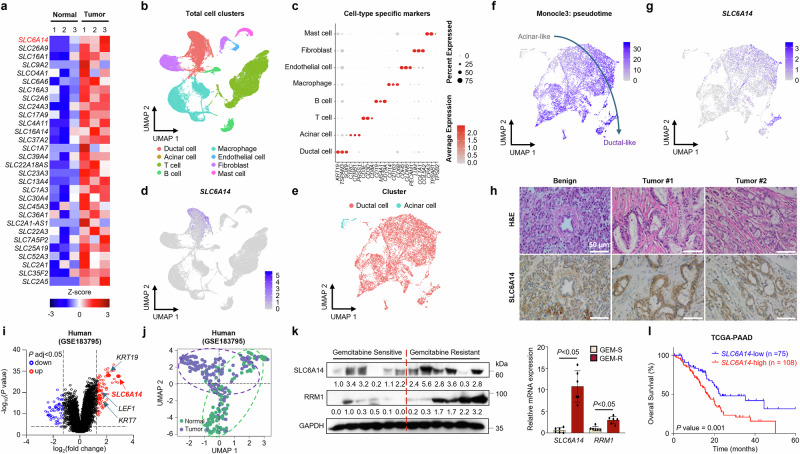
Overexpressed SLC6A14 in gemcitabine-resistant PDAC leads to a poor prognosis. **a** Heatmap of the *SLC* family population between normal and tumor cells at the RNA level. **b** UMAP plot of a total of eight major cell clusters in patients with PDAC (*n* = 23) **c** Dot plot of the top cell-type-specific markers for the patients with PDAC (*n* = 23). **d** Feature plots indicating the UMAP space in which cells were color-coded according to *SLC6A14* score. **e** UMAP plot of ductal and acinar cell clusters in patients with PDAC (n = 23). **f**, **g** Feature plots indicating the UMAP space in which cells were color-coded according to pseudotime score (**f**) and *SLC6A14* score (**g**). **h** H&E staining and immunohistochemistry assays were performed on normal pancreatic and PDAC tumors from patients (40× magnification). **i** Volcano plots. The log_2_(fold change) represents the mean expression level of each gene. Each dot represents a single gene. Black dots represent no significant DEGs between the normal and tumor groups, red dots represent upregulated genes and blue dots represent downregulated genes (GSE183795). **j** UMAP showing gene expression in normal pancreatic tissues and pancreatic cancer tissues (GSE183795). **k** Gemcitabine-sensitive (*n* = 6) and gemcitabine-resistant (*n* = 6), followed by western blot (left) and RT–qPCR (right) analysis. **l** Kaplan–Meier survival analyses of patients with PDAC, based on *SLC6A14* expression for overall survival. Scale bars, 50 μm (**h**). Error bars, mean ± s.d., ^#^*P* < 0.05, ^##^*P* < 0.01, ^###^*P* < 0.001; n.s., not significant; by Student’s *t*-test (**k**).

Quantitative mRNA analysis was performed to determine the differential expression of *SLC* family genes in pancreatic tumors compared with those in adjacent normal pancreatic tissues (Fig. 1a). Most *SLC* genes, including *SLC6A14*, were upregulated in PDAC tumors compared with those in adjacent normal tissues, with *SLC6A14* being the most differentially expressed gene (Fig. 1a and Supplementary Fig. 1a–c).

Single-cell RNA sequencing of 23 patients with PDAC (GSE155698 and GSE212966) identified eight distinct cell types: ductal cells, acinar cells, T cells, B cells, macrophages, endothelial cells, fibroblasts and mast cells (Fig. 1b). Cell types were verified using marker genes, such as *KRT19* for ductal cells, *CTRC* for acinar cells, *CD3* for T cells, *CD19* for B cells, *CD68* for macrophages, *CLDN5* for endothelial cells, *COL1A1* for fibroblasts and *CPA3* for mast cells (Fig. 1c and Supplementary Fig. 1d). *SLC6A14* was predominantly expressed in ductal cells and enriched in ductal-like tumors (Fig. 1d–g). Immunohistochemistry further confirmed elevated SLC6A14 expression in PDAC cells compared with adjacent normal cells (Fig. 1h). Microarray analysis revealed the differential expression of *SLC6A14* and oncogenic genes in PDAC tissues (Fig. 1i, j), with CAPAN-1 cells expressing the highest *SLC6A14* levels (Supplementary Fig. 1e).

To explore the role of SLC6A14 in gemcitabine resistance, we assessed ribonucleotide reductase M1 (RRM1) expression, a marker associated with resistance, which showed a positive correlation with gemcitabine-resistant PDAC samples^33^ (Fig. 1k). Gemcitabine resistance was defined as relapse within 1 year of treatment^34^. Drug-resistant PDAC cell lines, established via continuous gemcitabine exposure, displayed elevated SLC6A14 expression compared with sensitive lines, particularly CAPAN-1 (Supplementary Fig. 1f–i). Kaplan–Meier analysis indicated that high *SLC6A14* expression correlated with poorer overall and disease-specific survival in patients with PDAC (Fig. 1l and Supplementary Fig. 1j).

### Inhibition of SLC6A14 hinders EMT and cellular proliferation in conditions of glutamine deprivation and the presence of CSC

**Fig. 2:**
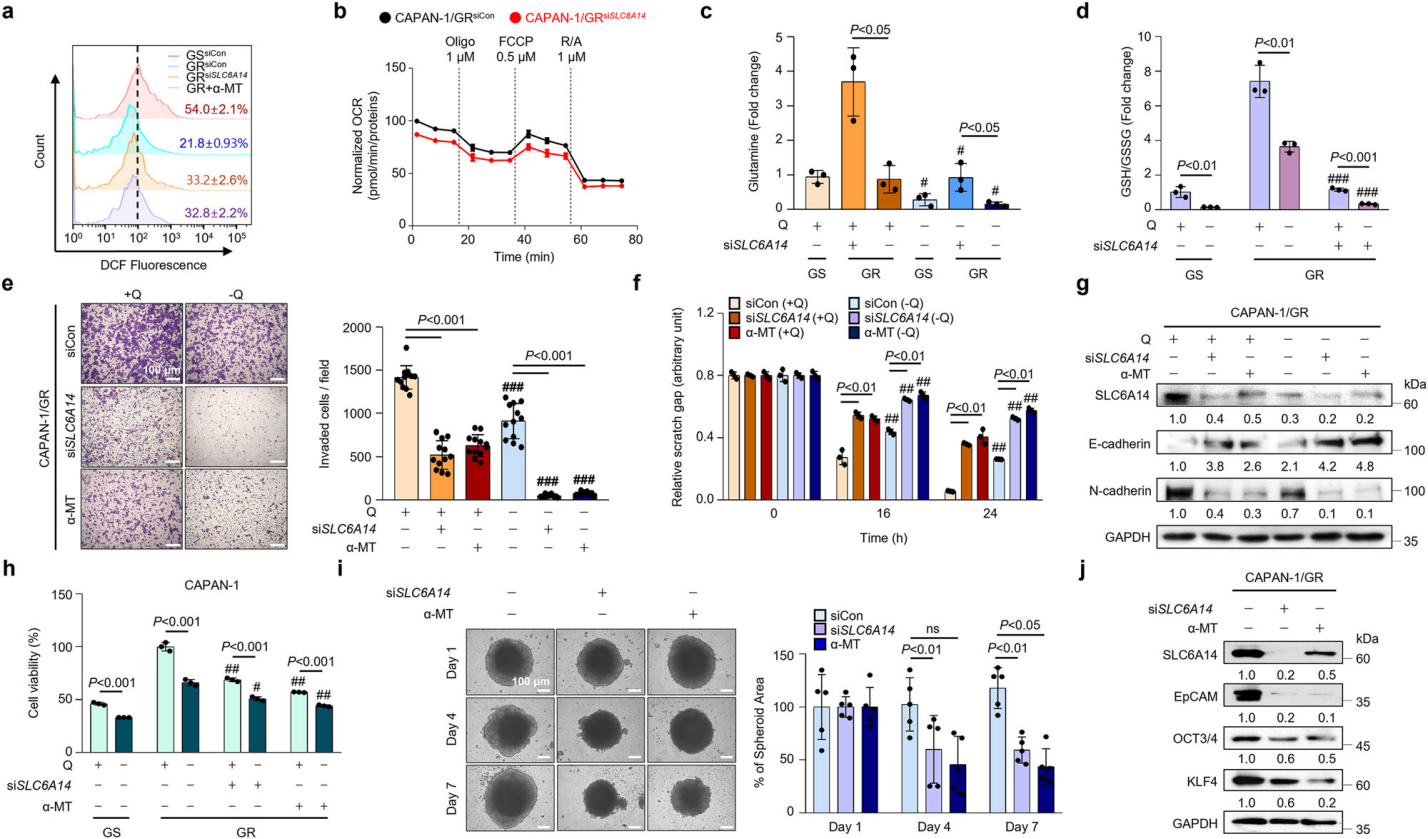
Inhibition of SLC6A14 hinders cellular metastasis in conditions of glutamine deprivation and the presence of CSCs. **a** ROS levels in CAPAN-1, CAPAN-1/GR and CAPAN-1/GR either transfected with si*SLC6A14* or treated with α-MT, measured by flow cytometry with DCF-DA staining. **b** OCR in CAPAN-1/GR and CAPAN-1/GR cells transfected with si*SLC6A14*. **c** The peak area of glutamine in CAPAN-1, CAPAN-1/GR si*Con* and CAPAN-1/GR si*SLC6A14* incubated for 24 h with a medium containing ±glutamine was measured by LC–MS analysis. **d** The GSH/GSSG ratio (GSSG, oxidized glutathione) in CAPAN-1, CAPAN-1/GR si*Con* and CAPAN-1/GR si*SLC6A14* incubated with ± glutamine for 24 h was measured by LC–MS analysis. **e** Invasion assay on CAPAN-1/GR si*Con*, CAPAN-1/GR si*SLC6A14* or α-MT for 24 h, then incubated with ±glutamine for 24 h (40× magnification). **f** Wound-healing assay on CAPAN-1/GR si*SLC6A14* or α-MT for 24 h, then incubated with ±glutamine for 24 h. **g** CAPAN-1/GR si*Con*, CAPAN-1/GR *siSLC6A14* or α-MT for 24 h, then incubated with ±glutamine for 24 h, followed by western blot analysis. **h** WST assay on CAPAN-1, CAPAN-1/GR si*Con*, CAPAN-1/GR si*SLC6A14* or α-MT for 24 h, then incubated with ±glutamine for 24 h. **i** Sphere formation assay on CAPAN-1/GR si*Con*, CAPAN-1/GR si*SLC6A14* or α-MT for 24 h, then visualized on the indicated time points (40× magnification). **j** Western blot analysis of stemness-related genes in CAPAN-1/GR si*Con*, and CAPAN-1/GR si*SLC6A14* or α-MT. Scale bars, 100 μm (**e** and **i**). Error bars, mean ± s.d., ^#^*P* < 0.05, ^##^*P* < 0.01; ^###^*P* < 0.001; n.s., not significant; by one-way ANOVA (**e**, **f** and **i**) or Student’s *t*-test (**c**, **d** and **h**).

Consistent with previous findings, silencing or inhibiting *SLC6A14* substantially reduced antioxidant levels, as indicated by decreased glutathione peroxidase 4 (*GPX4*) and nuclear factor erythroid-2-related factor 2 (*NRF2*), transcription factors regulating antioxidant responses, at both the mRNA and protein levels^35,36^ (Supplementary Fig. 2a,b). In CAPAN-1/GR cells, *SLC6A14* knockdown or inhibition with α-MT, a selective SLC6A14 inhibitor, increased reactive oxygen species (ROS) levels^24^ (Fig. 2a and Supplementary Fig. 2c) and reduced both the OCR and ECAR (Fig. 2b and Supplementary Fig. 2d–f).

Because glutamine metabolism supports antioxidant defenses, MS analysis revealed that *SLC6A14* depletion reduced glutamine and glutamate levels under glutamine-deprived conditions (Fig. 2c and Supplementary Fig. 2g). SLC6A14 blockade also decreased reduced glutathione (GSH) levels and increased oxidized GSH levels, further confirming impaired GSH homeostasis (Fig. 2d and Supplementary Fig. 2h,i).

Given the link between SLC6A14 and tumor progression, its role in metastasis was examined. Because epithelial–to–mesenchymal transition (EMT)-related transcription factors are known to promote chemoresistance^37^, this suggests that inhibiting *SLC6A14* may suppress EMT-driven drug resistance. To determine whether SLC6A14 regulates metastasis in gemcitabine-resistant PDAC, invasion and wound healing assays were performed. Glutamine deprivation and SLC6A14 inhibition markedly reduced cell motility and invasiveness (Fig. 2e, f, and Supplementary Fig. 2j–o). SLC6A14 blockade also increased the expression of E-cadherin, an epithelial marker, and decreased that of N-cadherin, a mesenchymal marker, indicating its role in EMT (Fig. 2g and Supplementary Fig. 2j–o).

Reducing glutamine availability or silencing *SLC6A14* also impaired cell proliferation in gemcitabine-resistant PDAC cell lines (AsPC-1, BxPC-3 and CAPAN-1) (Fig. 2h and Supplementary Fig. 2p,q).

In addition, SLC6A14 inhibition impaired sphere formation and reduced stemness-related gene expression, suggesting impaired cancer stemness (Fig. 2i, j and Supplementary Fig. 2r). Together, these findings highlight the role of SLC6A14 in supporting antioxidant defenses, metabolism, EMT and stemness in gemcitabine-resistant PDAC, underscoring its potential as a therapeutic target.

### Enhanced glutamine transport via SLC6A14 increases α-KG levels, activating mTOR/NF-κB signaling to upregulate SYTL4 and induce CXCL8 secretion

To investigate the metabolic changes induced by *SLC6A14* in gemcitabine-resistant cancer cells, we analyzed metabolic enzyme levels, including those involved in the tricarboxylic acid (TCA) cycle, in the absence of *SLC6A14* (Supplementary Fig. 3a, b). GSEA revealed that glutamate and α-KG pathway-related genes were scarcely enriched in *SLC6A14*-deficient gemcitabine-resistant cells compared with wild-type GR cells (Supplementary Fig. 3c,d). Many TCA cycle enzymes were upregulated in CAPAN-1/GR cells, while most TCA cycle enzymes, including glutaminase (*GLS*), were either partially unaffected or downregulated following *SLC6A14* knockdown (Supplementary Fig. 3a). *GLS* downregulation correlated with reduced glutamine metabolism, decreased intracellular GSH levels and increased ROS production, contributing to oxidative stress^38,39^. In addition, glutaminolysis-related enzymes, such as glutamate dehydrogenase 1 (*GLUD1*) and glutamic-oxaloacetic transaminase 2 (*GOT2*), were downregulated at the mRNA level under *SLC6A14* and glutamine deficiency (Supplementary Fig. 3e).

**Fig. 3:**
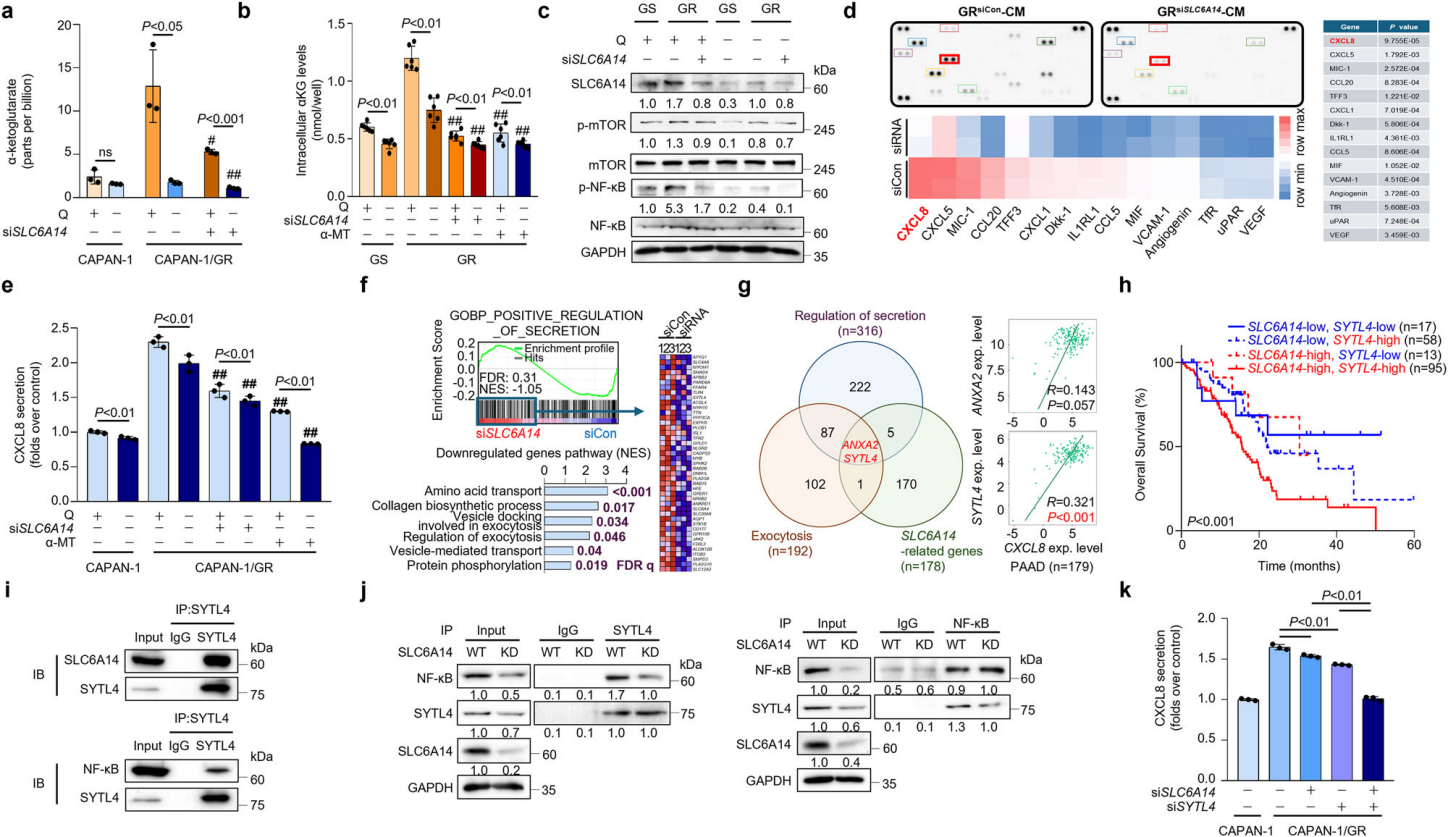
Enhanced glutamine transport via SLC6A14 increases α-KG levels, activating mTOR/NF-κB signaling to upregulate SYTL4 and induce CXCL8 secretion. **a** The peak area of α-KG in CAPAN-1, CAPAN-1/GR si*Con* and CAPAN-1/GR si*SLC6A14* incubated for 24 h with a medium containing ±glutamine was measured by LC–MS analysis. **b** Intracellular α-KG levels in CAPAN-1, CAPAN-1/GR si*Con* and CAPAN-1/GR si*SLC6A14* incubated for 24 h with a medium containing ±glutamine, followed by glutamine assays. **c** Western blot analysis of SLC6A14, p-mTOR, mTOR, p-NF-κB and NF-κB in CAPAN-1, CAPAN-1/GR si*Con* and CAPAN-1/GR si*SLC6A14* incubated for 24 h with ±glutamine. **d** Cytokine expression profiles in CAPAN-1/GR si*Con* versus si*SLC6A14* via cytokine assays. Cytokine detection (top left), heatmap (bottom left) and *P* values (right). **e** CXCL8 levels in CAPAN-1, CAPAN-1/GR si*Con*, CAPAN-1/GR si*SLC6A14* or α-MT for 24 h, then incubated with ±glutamine for 24 h. **f** GSEA of downregulated genes involved in the positive regulation of secretion in CAPAN-1/GR cells si*Con* versus si*SLC6A14* (FDR *q* value <0.05; top). KEGG enrichment analysis of genes downregulated in CAPAN-1/GR cells transfected with si*SLC6A14* (bottom). **g** A set of regulated secretion, exocytosis and *SLC6A14*-related genes were compared, and two common genes were identified. Correlation analyses of *CXCL8* between either *ANXA2* or *SYTL4* were performed using TISIDB. **h** Kaplan–Meier survival analysis of patients with PDAC based on *SLC6A14* and *SYTL4* expression for overall survival graph. **i** Endogenous SYTL4 was immunoprecipitated with endogenous SLC6A14 and NF-κB from CAPAN-1/GR cells and subjected to immunoprecipitation, followed by immunoblotting with indicated antibodies. **j** CAPAN-1/GR cells si*SLC6A14* were subjected to immunoprecipitation, followed by immunoblot analysis with indicated antibodies. **k** CXCL8 secretion levels in CAPAN-1, CAPAN-1/GR si*Con* and CAPAN-1/GR si*SLC6A14* or si*SYTL4*, and CAPAN-1/GR cells cotransfected with both si*SLC6A14* and si*SYTL4*. Error bars, mean ± s.d., ^#^*P* < 0.05; ^##^*P* < 0.01; ^###^*P* < 0.001; n.s., not significant; by Student’s *t*-test (**a**, **b**, **e** and **h**).

Given that *SLC6A14* depletion reduced glutamine and glutamate levels (Fig. 2c), we further examined α-KG levels. Both MS and ELISA confirmed that α-KG levels decreased with *SLC6A14* silencing under glutamine deprivation (Fig. 3a, b). TCA intermediates, including fumarate and malate, were also reduced, while aspartate and citrate levels remained unchanged (Supplementary Fig. 3f). Intracellular α-KG levels positively correlated with *SLC6A14* and glutamine levels (Fig. 3a, b) and are known to activate mTOR/NF-κB signaling^40,41^. We observed that mTORC1 and NF-κB phosphorylation were dependent on SLC6A14 and glutamine, indicating a role for α-KG in this regulatory axis (Fig. 3c and Supplementary Fig. 3g). Overall, our findings suggest that SLC6A14 modulates metabolic reprogramming in gemcitabine-resistant PDAC through glutaminolysis, amino acid metabolism and α-KG-mediated mTOR/NF-κB signaling.

Gemcitabine-resistant PDAC cells secreted elevated levels of CXCL8, a proinflammatory cytokine, compared with drug-sensitive cells (Fig. 3d and Supplementary Fig. 3h). Tumor–immune system interactions and drug bank (TISIDB) analysis confirmed a positive correlation between *SLC6A14* and *CXCL8*^42^ (Supplementary Fig. 3i). To assess the dependence of CXCL8 secretion on SLC6A14 and glutamine levels, we performed a CXCL8 ELISA under varying SLC6A14 levels and glutamine conditions (Fig. 3e). CAPAN-1/GR cells exhibited higher CXCL8 levels than CAPAN-1 cells, and glutamine depletion markedly reduced CXCL8 secretion. Furthermore, either *SLC6A14* silencing or inhibition of notably decreased CXCL8 secretion (Fig. 3e).

To identify the downstream targets influenced by *SLC6A14*, Gene Ontology analysis of RNA sequencing data comparing CAPAN-1/GR cells with and without *SLC6A14* depletion identified secretion- and exocytosis-related biological processes (Fig. 3f and Supplementary Fig. 3j). Among genes involved in secretion and exocytosis, *SYTL4* and annexin A2 (*ANXA2*) were substantially differentially expressed (Fig. 3g). High expression levels of *SLC6A14* and *SYTL4* correlated with poor overall survival in patients with PDAC (Fig. 3h). Correlation analysis revealed that *SYTL4* was correlated with *SLC6A14* and *RELA*, suggesting its involvement in NF-κB-mediated exocytosis (Supplementary Fig. 3k).

Further analysis demonstrated that SYTL4 directly interacts with SLC6A14 and binds to NF-κB (Fig. 3i). Silencing *SLC6A14* reduced the binding affinity between NF-κB and SYTL4, impairing cytokine secretion (Fig. 3j). SYTL4 expression was markedly higher in gemcitabine-resistant PDAC tumor tissues than in normal and gemcitabine-sensitive tissues (Supplementary Fig. 3l,m). *SYTL4* depletion reduced CXCL8 secretion similarly to *SLC6A14* knockdown (Fig. 3k) but did not noticeably affect mTOR/NF-κB phosphorylation levels (Supplementary Fig. 3n). These findings establish SYTL4 as a key downstream effector of SLC6A14, mediating CXCL8 secretion within the TME. Collectively, our findings highlight the *SLC6A14–SYTL4–CXCL8* axis as a potential therapeutic target in gemcitabine-resistant PDAC.

### CXCL8 secretion induced by glutamine initiates mitochondrial fission, rather than fusion, in CAFs, facilitating the upregulation of glutamine production

**Fig. 4:**
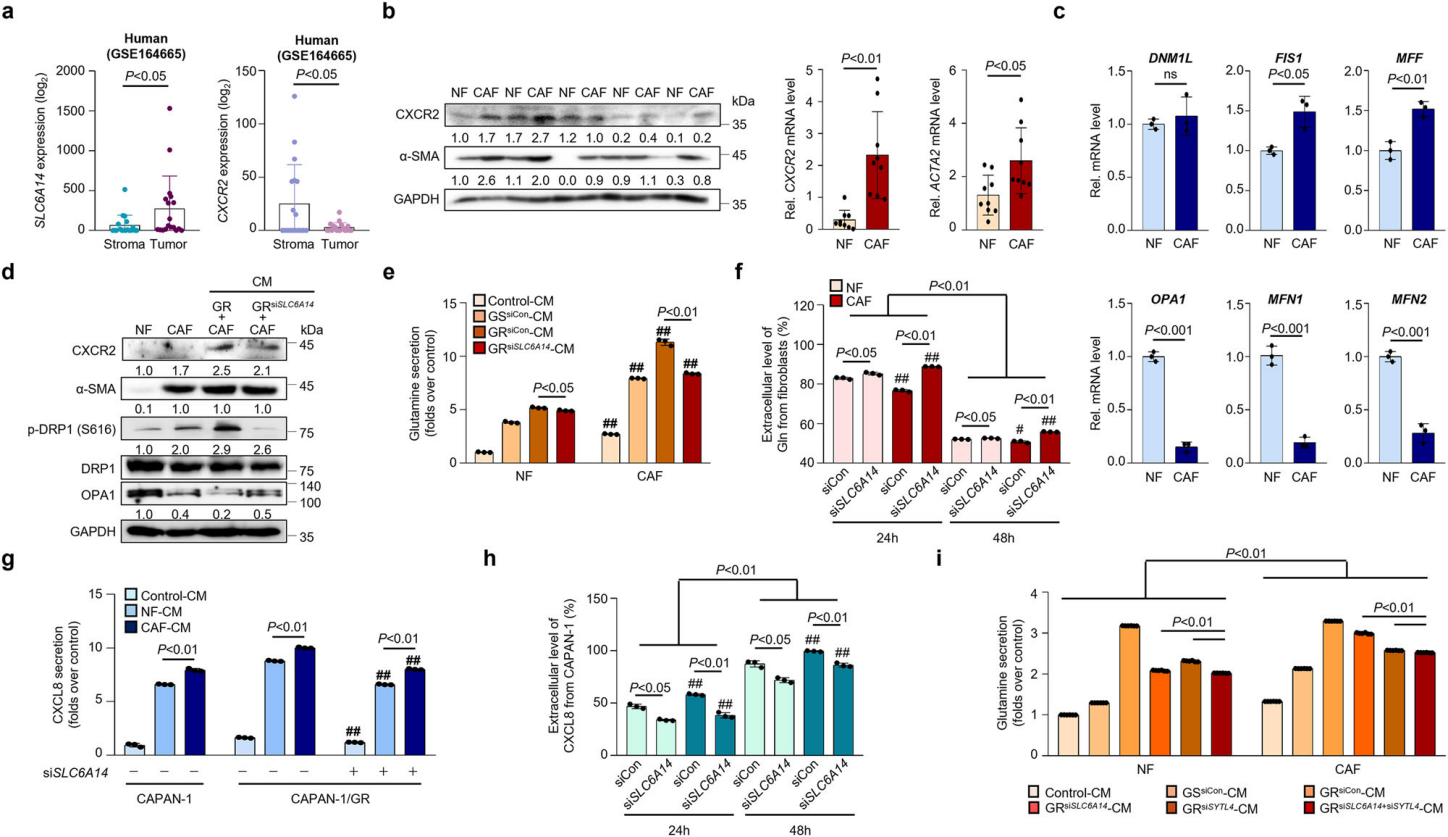
CXCL8 secretion induced by glutamine initiates mitochondrial fission, rather than fusion, in CAFs, facilitating the upregulation of glutamine production. **a** *SLC6A14* and *CXCR2* gene expression in the stroma (*n* = 18) versus tumor (*n* = 18) tissues from patients with PDAC (GSE164665). **b** CXCR2 and α-SMA levels in NFs and CAFs via western blot analysis. **c** Expression of mitochondrial fission genes (*DNM1L*, *FIS1* and *MFF*) and fusion genes (*OPA1*, *MFN1* and *MFN2*) in NF and CAF via qRT–PCR analysis. **d** Western blot analysis of α-SMA, p-DRP1, DRP1 and OPA1 in NF, CAF and CAF incubated with CAPAN-1/GR si*Con* or si*SLC6A14*-conditioned medium for 24 h. **e** Glutamine secretion in NF and CAF cocultured with control, CAPAN-1, CAPAN-1/GR si*Con* and CAPAN-1/GR si*SLC6A14*-conditioned medium for 24 h. **f** Secreted extracellular glutamine in NF or CAF cocultured with CAPAN-1/GR si*Con* or si*SLC6A14*-conditioned medium at 24 and 48 h, calculated by subtracting the initial time point from the consecutive time points. **g** CXCL8 levels in control, CAPAN-1, CAPAN-1/GR si*Con* and CAPAN-1/GR si*SLC6A14* cocultured with NF and CAF-conditioned medium for 24 h. **h** Secreted extracellular CXCL8 from CAPAN-1/GR si*Con* versus si*SLC6A14* cocultured with NF- or CAF-conditioned medium at 24 and 48 h, calculated by subtracting the initial time point from the consecutive time points. **i** Glutamine secretion levels of NF and CAF followed by 24 h coculture with control, CAPAN-1, CAPAN-1/GR si*Con*, CAPAN-1/GR si*SLC6A14* or si*SYTL4*, and CAPAN-1/GR both si*SLC6A14* and si*SYTL4*. Error bars, mean ± s.d., ^#^*P* < 0.05, ^##^*P* < 0.01, ^###^*P* < 0.001; n.s., not significant; by one-way ANOVA (**f**, **h** and **i**) or Student’s *t*-test (**a**–**c**, **e** and **g**).

Transcriptomic analysis of the microdissected tumors (GSE164665, Fig. 4a) revealed higher expression of *CXCR2*, a known receptor for CXCL8, in stromal cells, whereas *SLC6A14* was predominantly expressed in cancer cells (Fig. 4a). CAFs exhibited markedly higher *CXCR2* mRNA and protein levels than NFs, along with increased α-smooth muscle actin (α-SMA) expression (Fig. 4b and Supplementary Fig. 4a,b).

Given the established CXCL8–PI3K–AKT signaling axis^43,44^, we hypothesized that CXCL8 activates CXCR2 in fibroblasts, initiating PI3K/AKT signaling and promoting mitochondrial fission via dynamin-related protein 1 (DRP1) phosphorylation. CAFs displayed enhanced PI3K/AKT signaling, increased DRP1 phosphorylation and reduced optic atrophy 1 (OPA1) expression (Supplementary Fig. 4c). Furthermore, CAFs upregulated mitochondrial fission genes (*Fis1* and *MFF*) and downregulated mitochondrial fusion genes (*OPA1*, *MFN1* and *MFN2*) (Fig. 4c). Coculturing CAFs with drug-resistant PDAC-conditioned medium further increased phosphorylated DRP1 levels, an effect that was diminished by *SLC6A14* silencing (Fig. 4d).

To investigate the role of glutamine in gemcitabine-resistant PDAC within the TME, we focused on fibroblast-mediated nutrient exchange. Gemcitabine-resistant cells rely on glutamine for survival, and CAFs release higher levels of glutamine than NFs. Notably, SLC6A14 inhibition reduced glutamine secretion in both fibroblast types (Fig. 4e and Supplementary Fig. 4d,e). Coculture experiments showed that fibroblasts and GR-PDAC progressively increased the extracellular glutamine levels over time, indicating that fibroblasts contributed to the glutamine pool (Fig. 4f and Supplementary Fig. 4f).

In addition to metabolic interactions, CAF-conditioned medium induced greater CXCL8 release from cancer cells than NF-conditioned medium (Fig. 4g and Supplementary Fig. 4g). *SLC6A14* silencing in CAPAN-1/GR cells notably reduced CXCL8 secretion over time (Fig. 4h). SYTL4 mediates CXCL8 secretion from gemcitabine-resistant cancer cells; therefore, we further examined its role in fibroblast–glutamine exchange. Dual silencing of *SLC6A14* and *SYTL4* led to the greatest reduction in glutamine secretion by fibroblasts (Fig. 4i). These findings highlight the critical role of SLC6A14 in glutamine uptake, cytokine secretion and fibroblast interactions within the TME, thereby providing insights into PDAC nutrient dynamics and potential therapeutic targets.

### SLC6A14 attenuates T cell infiltration through PD-L1 regulation

**Fig. 5:**
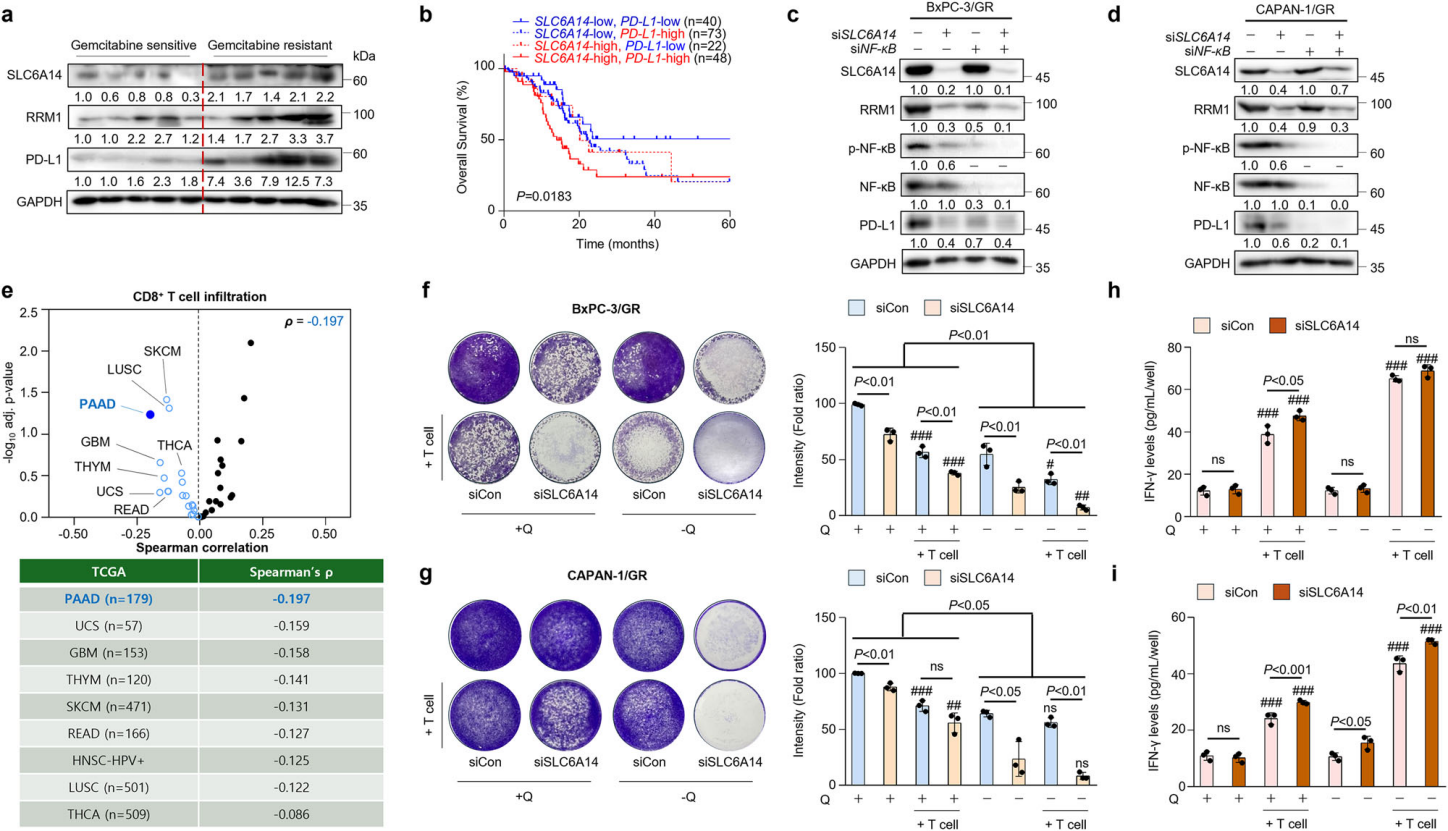
SLC6A14 attenuates T cell infiltration through PD-L1 regulation. **a** Gemcitabine-sensitive (*n* = 5) and gemcitabine-resistant (*n* = 5) cells were analyzed by western blot. **b** Kaplan–Meier survival analysis of patients with PDAC based on *SLC6A14* and *PD-L1* expression for overall survival graph. **c** Expression of SLC6A14, RRM1, p-NF-κB, NF-κB and PD-L1 in BxPC-3/GR si*Con*, BxPC-3/GR si*SLC6A14* or si*NF-κB*, and BxPC-3/GR cells cotransfected with both si*SLC6A14* and si*NF-κB*, analyzed by western blot. **d** Expression of SLC6A14, RRM1, p-NF-κB, NF-κB and PD-L1 in CAPAN-1/GR si*Con*, CAPAN-1/GR si*SLC6A14* or si*NF-κB*, and CAPAN-1/GR cells cotransfected with both si*SLC6A14* and si*NF-κB* via western blot analysis. **e** Volcano plot of the Spearman correlation between CD8^+^ T cell infiltration and SLC6A14 in 40 cancer types. Blue points indicate cancer types in which SLC6A14 was significantly negatively correlated with CD8^+^ T cell infiltration (adjusted *P* < 0.05). **f**, **g** T cell-mediated cancer cell killing. The GR-PDAC cell lines, BxPC-3/GR (**f**) and CAPAN-1/GR (**g**), with si*SLC6A14* incubated for 24 h with ±glutamine were cocultured with activated human CD8^+^ T cells for 48 h to observe the effects of SLC6A14 on T cells. The cancer cells that survived were stained with crystal violet. The ratio of cancer cells to T cells was 1:10. Representative images are shown on the left, and quantitative data are shown on the right. **h**, **i** Activated CD8^+^ T cells cocultured with BxPC-3/GR (**h**) and CAPAN-1/GR (**i**) cells subjected to ELISA for IFN-γ measurement. Error bars, mean ± s.d., ^#^*P* < 0.05, ^##^*P* < 0.01, ^###^*P* < 0.001; n.s., not significant; by one-way ANOVA (**f** and **g**) or Student’s *t*-test (**h** and **i**).

To examine whether SLCA14 regulates immune cell infiltration, we assessed PD-L1 expression in PDAC tumor tissues and adjacent normal pancreatic tissues. PD-L1 expression was markedly higher in PDAC tumors than in normal tissues (Supplementary Fig. 5a). Furthermore, PD-L1 levels were higher in gemcitabine-resistant than in gemcitabine-sensitive PDAC (Fig. 5a and Supplementary Fig. 5b), suggesting a positive correlation between SLC6A14 and PD-L1 expression. PD-L1 expression in cancer cells increased upon nutrient supplementation, whereas it was reduced following *SLC6A14* silencing or glutamine deprivation (Supplementary Fig. 5c). Kaplan–Meier survival analysis revealed that high expression levels of both *SLC6A14* and *PD-L1* were associated with poor overall survival in patients with PDAC (Fig. 5b).

A previous study demonstrated that NF-kB regulates PD-L1 expression in cancers^45,46^. To investigate whether NF-kB interacts with PD-L1 in gemcitabine-resistant cancer cells, we assessed PD-L1 protein levels under SLC6A14 and NF-kB regulation. PD-L1 expression was downregulated upon silencing either *SLC6A14* or *NF-kB* (Fig. 5c, d).

Moreover, analysis of the TCGA dataset showed that patients with pancreatic cancer exhibited lower CD8^+^ T cell infiltration than those with other cancer types (Fig. 5e and Supplementary Fig. 5d, e). Given that SLC6A14 regulates PD-L1 expression in gemcitabine-resistant PDAC, we examined its role in immune evasion during *SLC6A14* or glutamine deprivation. Notably, gemcitabine-resistant PDAC cells were protected from T cell infiltration due to SLC6A14-mediated upregulation of PD-L1. However, upon *SLC6A14* or glutamine deprivation, these cells became more susceptible to T cell infiltration and were markedly impaired under nutrient-deprived conditions (Fig. 5f, g). Finally, CD8^+^ T cells exhibited notably increased activation, as indicated by elevated IFN-γ expression, when both glutamine and *SLC6A14* were deficient (Fig. 5h, i). These findings indicate that SLC6A14 promotes immune evasion not only by inducing PD-L1 expression but also by functionally suppressing CD8⁺ T cell effector activity. The restoration of IFN-γ production upon *SLC6A14* knockdown suggests that its inhibition reverses the immunosuppressive phenotype of gemcitabine-resistant PDAC cells, thereby reactivating antitumor T cell responses.

### Depletion of SLC6A14 sensitizes and attenuates tumor–fibroblast complexes to gemcitabine, suppressing metastasis, tumorigenesis and fibroblast activation in vivo

**Fig. 6:**
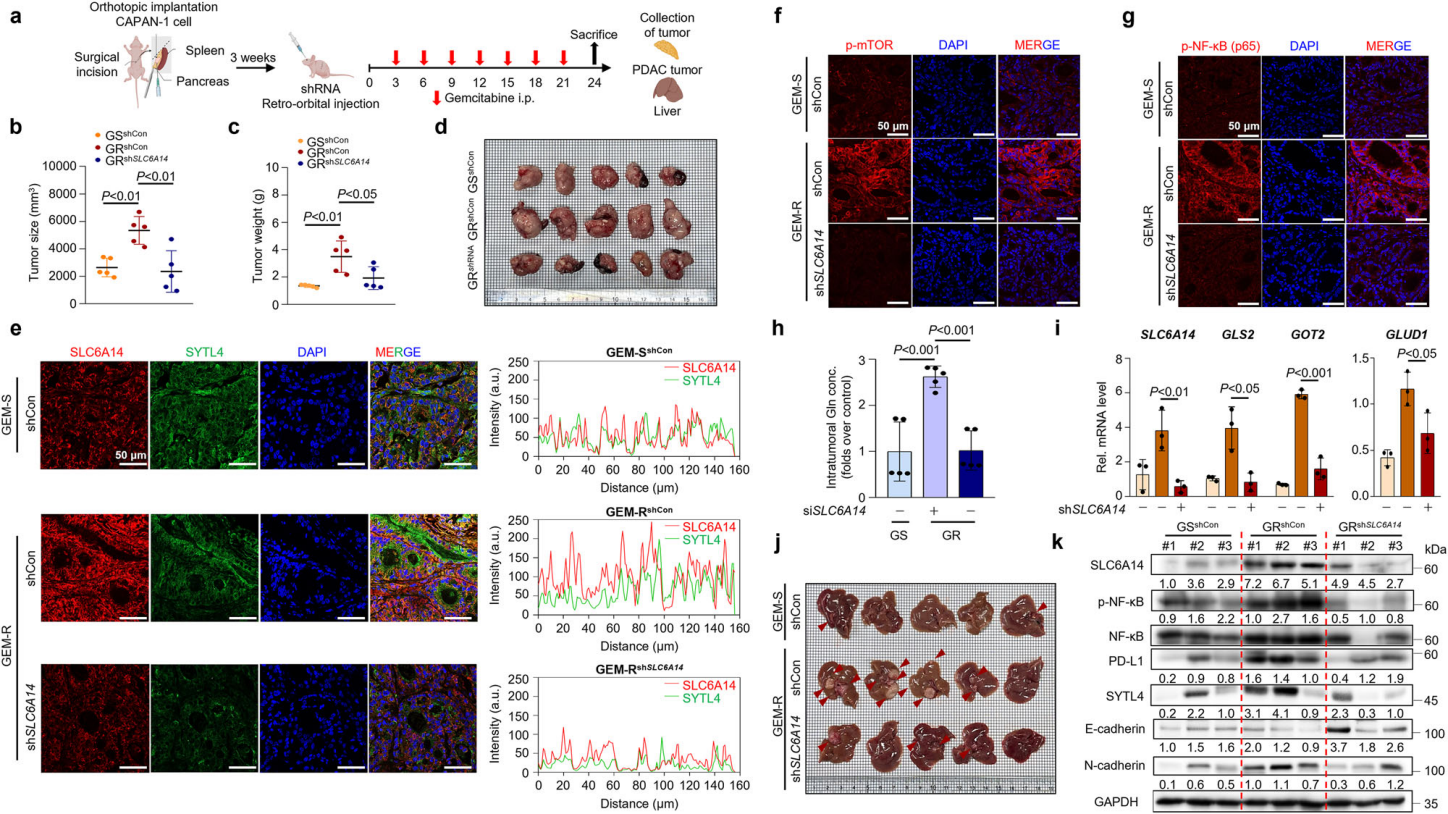
Blockade of SLC6A14 inhibits glutamine production suppressing tumorigenesis and liver metastasis. **a** BALB/c nude mice were orthotopically injected with CAPAN-1 or CAPAN-1/GR cells (3 × 10^6^). After 3 weeks, lentivirus particles carrying sh*Control* or sh*SLC6A14* were injected (2 × 10^6^ particles) via the retro-orbital route. After 1 week, 10 mg/kg gemcitabine (*n* = 5) was administered intraperitoneally twice a week. **b** Tumor size. **c** Tumor weight. **d** Representative images of pancreatic tumors (*n* = 5). **e** Immunofluorescence staining of SLC6A14 (red), SYTL4 (green) and DAPI (blue) for the nuclei. **f** Immunofluorescence staining of p-mTOR (red) and DAPI (blue) in the nuclei. **g** Immunofluorescence staining of p-NF-κB (red) and DAPI (blue) in the nuclei. **h** Intratumoral glutamine levels in the pancreatic tissue. **i** Expression levels of *SLC6A14*, *GLS*, *GOT2* and *GLUD1* in the pancreatic tissues, followed by qRT–PCR analysis. **j**, Liver metastases (*n* = 5). **k** Expression of SLC6A14, p-NF-κB, NF-κB, PD-L1, SYTL4, E-cadherin and N-cadherin in pancreatic tissues, followed by western blotting analysis. Scale bars, 50 μm (**e**–**g**). Error bars, mean ± s.d., ^#^*P* < 0.05, ^##^*P* < 0.01, ^###^*P* < 0.001; n.s., not significant; by one-way ANOVA (**b**, **c** and **h**) or Student’s *t*-test (**i**). GS-sh*Con*, gemcitabine-sensitive sh*Control*; GR-sh*Con*, gemcitabine-resistant sh*Control*; GR-sh*SLC6A14*, gemcitabine-resistant sh*SLC6A14*.

To investigate the role of SLC6A14 in metastasis and tumorigenesis, orthotopic xenograft models were constructed by implanting CAPAN-1 and CAPAN-1/GR cells into nude mice with or without *SLC6A14* inhibition (Fig. 6a). *SLC6A14* inhibition noticeably reduced tumor growth and weight without affecting body weight (Fig. 6b–d and Supplementary Fig. 6a). H&E staining revealed less aggressive tumor morphology in *SLC6A14*-depleted gemcitabine-resistant tumors (Supplementary Fig. 6b). Immunofluorescence revealed prominent colocalization of SLC6A14 and SYTL4 in GR-PDAC, which decreased upon *SLC6A14* silencing (Fig. 6e). Levels of phosphorylated mTOR, NF-κB, PD-L1 and Ki-67 (a marker of tumor proliferation) were also reduced following *SLC6A14* inhibition (Fig. 6f, g and Supplementary Fig. 6c). *SLC6A14* inhibition lowered glutaminolysis-related enzyme mRNA levels and glutamine levels in PDAC tissues (Fig. 6h, i).

Metastatic potential was assessed by counting liver metastases (Fig. 6j). Liver metastases were prevalent in gemcitabine-resistant models, but markedly reduced by *SLC6A14* inhibition, with no lung metastases observed (Supplementary Fig. 6d–h). Protein levels of SLC6A14, NF-κB, PD-L1, SYTL4, E-cadherin and N-cadherin were consistent with previous models (Fig. 6k). These findings suggest SLC6A14 regulates glutamine levels and liver metastasis in GR-PDAC.

**Fig. 7:**
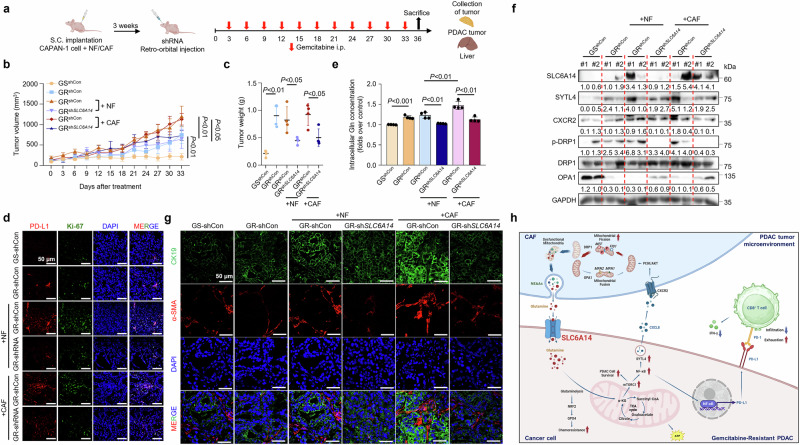
Depletion of SLC6A14 attenuates fibroblast activation and tumor growth in tumor–fibroblast complexes. **a** BALB/c nude mice were subcutaneously injected with CAPAN-1 or CAPAN-1/GR cells (2 × 10^5^) with or without NFs or CAFs (3 × 10^5^). After 3 weeks, lentivirus particles carrying sh*Control* or sh*SLC6A14* were injected (2 × 10^6^ particles) via the retro-orbital route. After 1 week, 10 mg/kg gemcitabine (*n* = 5) was administered intraperitoneally twice a week. **b** Tumor volume. **c** Tumor weight. **d** Immunofluorescence staining of PD-L1 (red), ki-67 (green) and DAPI (blue) in the nuclei. **e** Intratumoral glutamine levels, followed by glutamine assays. **f** Protein expression of SLC6A14, SYTL4, CXCR2, p-DRP1, DRP1 and OPA1 in the pancreatic tissues. **g** Immunofluorescence staining of CK19 (green), α-SMA (red) and DAPI (blue) in the nuclei. **h** Schematic representation of the mechanism by which SLC6A14 confers gemcitabine resistance in pancreatic cancer by upregulating NF-κB/PD-L1 to promote immune evasion and activating SYTL4/CXCL8–CXCR2 signaling to reprogram the gemcitabine-resistant TME. Scale bars, 50 μm (**d** and **g**). Error bars, mean ± s.d., ^#^*P* < 0.05, ^##^*P* < 0.01, ^###^*P* < 0.001; n.s., not significant; by one-way ANOVA (**b** and **e**) or Student’s *t*-test (**c**).

To assess the role of SLC6A14 in tumor–fibroblast interactions, CAPAN-1 cells were subcutaneously injected into mice (Fig. 7a). Gemcitabine-resistant tumors grew larger than sensitive models (Fig. 7b). Co-injection with CAFs further increased tumor growth, which was reduced by *SLC6A14* inhibition (Fig. 7c, d and Supplementary Fig. 7a, b). *SLC6A14* silencing in GR cells with fibroblast co-injection attenuated proliferation, with no liver metastasis observed (Supplementary Fig. 7c). *SLC6A14* knockdown reduced glutaminolysis enzyme mRNA levels (Supplementary Fig. 7d) and reversed CAF-induced glutamine increases (Fig. 7e).

Western blotting revealed reduced mitochondrial fission following *SLC6A14* inhibition (Fig. 7f). The expression levels of PDAC (CK19) and fibroblast (α-SMA) markers were highest in GR cells co-injected with CAFs, decreasing upon *SLC6A14* disruption (Fig. 7g). These results highlight the key role of SLC6A14 in metastasis and tumor growth, as well as its potential as a therapeutic target in GR-PDAC.

## Discussion

Our study identifies SLC6A14 as a pivotal regulator of gemcitabine resistance and immune evasion in PDAC through its effects on glutamine metabolism, immune checkpoint signaling and tumor–stroma interactions. Targeting SLC6A14 may overcome chemoresistance and improve antitumor immunity in PDAC.

Glutamine metabolism is closely linked to drug resistance in various cancers^47,48^. In breast cancer, glutamine uptake induces tamoxifen resistance via the PI3K–AKT–PTEN pathway^49^, while glutaminolysis contributes to CDK4/6 inhibitor resistance in esophageal squamous cancer^50^. Zhou et al. also reported that glutamine dependency drives apatinib resistance via the GCN2–eIF2α–ATF4 pathway in non-small-cell lung cancer^51^. Although pancreatic cancer relies heavily on glutamine^52^, the precise mechanisms underlying its dependency remain unclear. Our findings demonstrate that *SLC6A14* facilitates glutamine uptake and conversion to α-KG, a key metabolite that activates mTORC1 signaling^38,41,53^. Inhibition of SLC6A14 disrupted glutaminolysis, impaired α-KG production, suppressed mTORC1/NF-κB signaling and induced autophagy (Supplementary Fig. 3g), highlighting its role in gemcitabine resistance. Mechanistically, our data support a sequential signaling cascade in which SLC6A14-mediated glutamine uptake increases intracellular levels of glutamate and α-KG, promoting mTORC1 activation—probably through Rag-GTPase-dependent recruitment to the lysosomal membrane^40^. Subsequent mTORC1 activation enhances the kinase activities of IKKα and IKKβ, leading to NF-κB phosphorylation and activation. Activated NF-κB, in turn, promotes the transcription of immune-modulatory genes^41,54,55^.

Among these, PD-L1 was upregulated in a glutaminolysis-dependent manner, contributing to immune evasion. To further explore how SLC6A14 affects T cell-mediated immunity, we conducted CD8⁺ T cell coculture and IFN-γ ELISA assays. Silencing *SLC6A14* in gemcitabine-resistant PDAC cells markedly enhanced T cell cytotoxic activity and restored IFN-γ secretion, consistent with reduced PD-L1 expression. These findings suggest that SLC6A14 not only regulates PD-L1 transcription via metabolic signaling but also suppresses T cell effector functions in the TME. Therefore, SLC6A14 inhibition may serve as a strategy to reverse immune evasion and enhance immunotherapy responses in PDAC. Gopal et al. demonstrated that targeting GLS with CB-839 in melanoma cells, in combination with anti-PD-1 and anti-CTLA4 antibodies, enhanced the therapeutic efficacy of immune checkpoint blockade (ICB)^56^. Similarly, a study investigating the arginase inhibitor, CB-1158, in combination with PD-L1 blockade, showed promising results in inhibiting cancer growth^57^. Building on prior studies demonstrating the synergy between metabolic inhibitors and ICB, our findings suggest that SLC6A14 inhibition could potentiate ICB efficacy in gemcitabine-resistant PDAC. Interestingly, gemcitabine-resistant PDAC tumors displayed concurrent upregulation of Ki-67 and PD-L1 (Fig. 7d and Supplementary Fig. 6c), suggesting an adaptive proliferative response alongside immune modulation. Given that PD-L1 expression is associated with improved ICB responses^58,59^, targeting SLC6A14 in combination with α-MT and glutamine blockade may offer a promising strategy to improve antitumor responses.

Our investigation into the secretome of gemcitabine-resistant PDAC cells revealed elevated CXCL8 levels, a chemokine known to support tumor growth and chemoresistance. Silencing *SLC6A14*-dependent mTORC1/NF-κB signaling was found to reduce CXCL8 secretion via SYTL4, a key regulator of exocytic vesicle transport, as confirmed by ELISA^60–64^ (Fig. 3e). SYTL4-mediated exocytosis facilitated CXCL8 release from gemcitabine-resistant PDAC cells (Fig. 3k). Because the TME plays a multifaceted role in enhancing oncogenicity and fostering malignancy, particularly in PDAC^65^, cancer cells engage in competitive nutrient acquisition, which often results in nutrient deprivation in the surrounding stroma^66^. Yang et al. demonstrated that ovarian cancer cells promote glutamine production in CAFs through the uptake of asparagine, aspartate and glutamate, thus establishing a reciprocal exchange of glutamate and glutamine between cancer cells and CAFs^38,67^. In addition, Chen et al. reported that tumor cells and myofibroblasts interact via homophilic ATP1A1 bands, triggering activin A secretion, which enhances tumor invasiveness and metastasis^13^. Consequently, tumor–fibroblast interactions support chemoresistance through metabolic reprogramming via juxtacrine or paracrine signaling. Given that CXCL8 enhances fibroblast activation via CXCR2 signaling, we propose that SLC6A14 regulates tumor–stroma interactions by sustaining CXCL8 secretion.

The mechanism by which CXCL8 signaling induces mitochondrial fission and increases glutamine production in CAFs remains unclear. Upon paracrine CXCL8 signaling, CXCR1/2 receptors are internalized, with CXCR2 being internalized more rapidly than CXCR1^68^. Cai et al. reported that mitochondrial fission has been linked to the mTOR–DRP1 axis in fibroblasts under glutamine-depleted conditions in renal cancer^69^. To investigate whether CXCR2 mediates PI3K/AKT signaling during fibroblast activation and to explore tumor–stroma metabolic interactions, we isolated primary fibroblasts from resected human PDAC tissues and expanded in culture. Transcriptomic and protein analyses revealed that these fibroblasts exhibited high expression of canonical myCAF markers, *ACTA2* and *POSTN*, and minimal expression of iCAF- or apCAF-associated genes, such as *IL6*, *LIF*, *PDGFRA*, *CD74* and *HLA-DRA* (Fig. 4b and Supplementary Fig. 4a,b). This expression profile is consistent with a myCAF phenotype, typically characterized by contractility and proximity to cancer cells. We next examined AKT phosphorylation at serine 473 (Ser473), a known activator of DRP1-mediated mitochondrial fission in CAFs^43,44^. Our analysis of mitochondrial dynamics revealed a notable upregulation of mitochondrial fission-associated genes following CXCL8–CXCR2 activation. Mitochondrial fission is known to facilitate the proteasomal degradation of damaged mitochondrial components, generating amino acids that contribute to cellular metabolism. We observed that CAFs produced substantially higher amounts of glutamine than NFs. Importantly, silencing *SLC6A14* inhibited CXCL8/CXCR2 signaling, leading to a reduction in mitochondrial fission and subsequent glutamine production in fibroblasts. This suggests that SLC6A14-mediated CXCL8 regulates mitochondrial dynamics to enhance nutrient availability in the surrounding TME. Given that cancer cells often deplete extracellular nutrients, CAFs appear to engage compensatory mechanisms to maintain metabolic balance. Zhang et al. reported that glutamine deprivation in the TME triggers calcium-dependent CaMKK2/ARHGEF2 signaling, driving macropinocytosis-mediated albumin uptake in CAFs, which is subsequently processed into non-essential amino acids to sustain cancer cell growth^70^. These findings underscore the complex metabolic interplay between CAFs and cancer cells, wherein CAFs activate diverse pathways to sustain their metabolic demands and promote tumor progression^70,71^ (Fig. 7h).

In conclusion, our findings establish SLC6A14 as a critical modulator of metabolic adaptation and immune evasion in gemcitabine-resistant PDAC. By regulating α-KG/NF-κB/PD-L1 and SYTL4/CXCL8–CXCR2 signaling, SLC6A14 drives both chemoresistance and immune suppression. Targeting SLC6A14, either alone or in combination with glutamine metabolism inhibitors and ICB therapy, may offer a promising strategy to improve treatment outcomes in glutamine-dependent pancreatic cancer.

## Supplementary information

Supplementary Information
