# Supplementary material for: SLC6A14-mediated glutamine promotes SYTL4–CXCL8 axis activation to drive gemcitabine resistance and immune evasion in pancreatic cancer
Source: Exp Mol Med. 2025 Dec 25;57(12):2943–56. doi: 10.1038/s12276-025-01596-w (PMC12800172; doi:10.1038/s12276-025-01596-w)
Supplement: Supplementary file 1 — Supplementary Information [file 12276_2025_1596_MOESM1_ESM.pdf]

Supplementary Figures

Supplementary Fig. 1

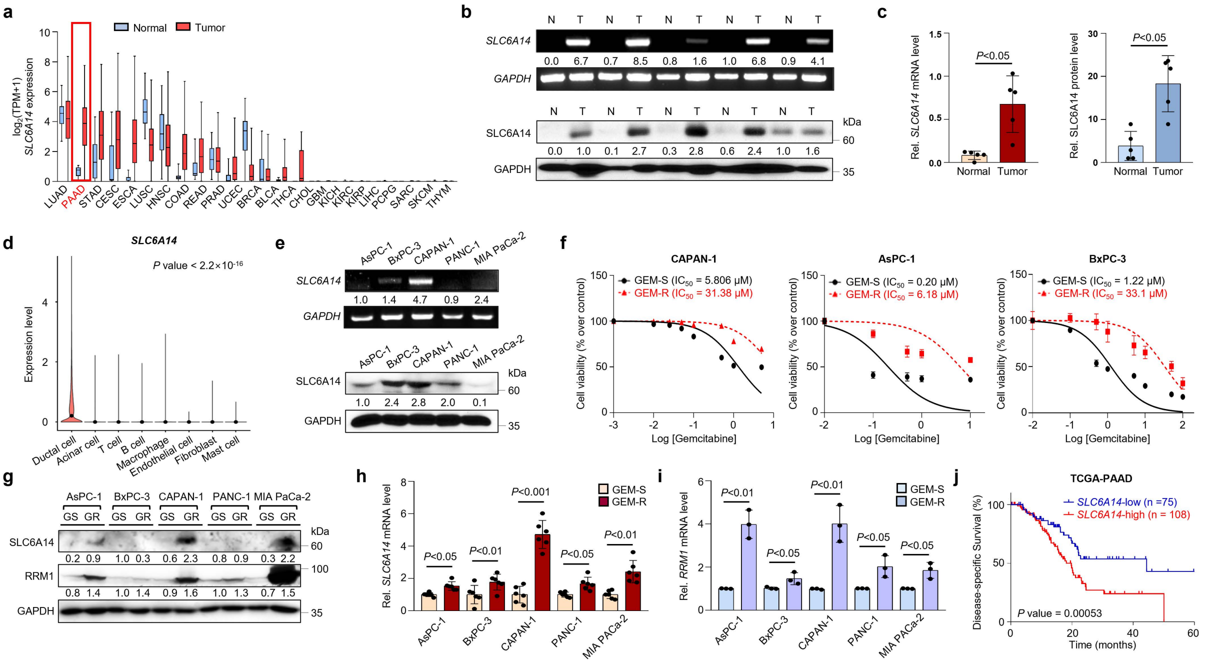

Supplementary Fig. 1. SLC6A14 exhibits higher upregulation in gemcitabine-resistant pancreatic cancer cell lines compared to gemcitabine-sensitive cancer cell lines.

a Differential expression of SLC6A14 in tumor and normal tissues. LUAD, lung adenocarcinoma; PAAD, pancreatic adenocarcinoma; STAD, stomach adenocarcinoma; CESC, cervical squamous cell carcinoma, and endocervical adenocarcinoma; ESCA, esophageal carcinoma; LUSC, lung squamous cell carcinoma; HNSC, head, and neck squamous cell carcinoma; COAD, colon adenocarcinoma; READ, rectum adenocarcinoma; PRAD, prostate adenocarcinoma; UCEC, uterine corpus endometrial carcinoma; BRCA, breast invasive carcinoma; BLCA, bladder urothelial carcinoma; THCA, thyroid carcinoma; CHOL, cholangiocarcinoma; GBM, glioblastoma multiforme; KICH, kidney chromophobe; KIRC, kidney renal clear cell carcinoma; KIRP, kidney renal papillary cell carcinoma; LIHC, liver hepatocellular carcinoma; PCPG, pheochromocytoma and paraganglioma; SARC, sarcoma; SKCM, skin cutaneous melanoma; THYM, thymoma. **b**, **c** SLC6A14 expression levels in normal pancreatic (n = 5) and PDAC tissue samples (n = 5), followed by RT-PCR and western blotting (**b**) and qRT-PCR and protein densitometry analysis (**c**). **d** Violin plot of a total of 8 major cell clusters in PDAC patients (n = 23). **e** SLC6A14 expression in AsPC-1, BxPC-3, CAPAN-1, PANC-1, and MIA PaCa-2 cell lines, followed by RT-PCR (upper) and western blotting analysis (lower). **f** IC_{50} values of gemcitabine in CAPAN-1/GS, CAPAN-1/GR, AsPC-1/GS, AsPC-1/GR, BxPC-3/GS, and BxPC-3/GR cells were determined using a non-linear regression model. **g** Expression levels of SLC6A14 and RRM1 in AsPC-

1, BxPC-3, CAPAN-1, PANC-1, and MIA PaCa-2 gemcitabine-sensitive and gemcitabine-resistant pancreatic cancer cell lines, followed by western blotting analysis. **h**, **i** SLC6A14 (**h**) and RRM1 (**i**) expression levels in gemcitabine-sensitive and gemcitabine-resistant PDAC cell lines, followed by qRT-PCR analysis. **j** Kaplan–Meier survival analyses of patients with PDAC, based on SLC6A14 expression for disease-specific survival. Error bars, mean ± SD, #p < 0.05; ##p < 0.01; ###p < 0.001; n.s., not significant; by Kruskal-Wallis test (**d**) or Student's t test (**c**, **h**, **i**).

Supplementary Fig. 2

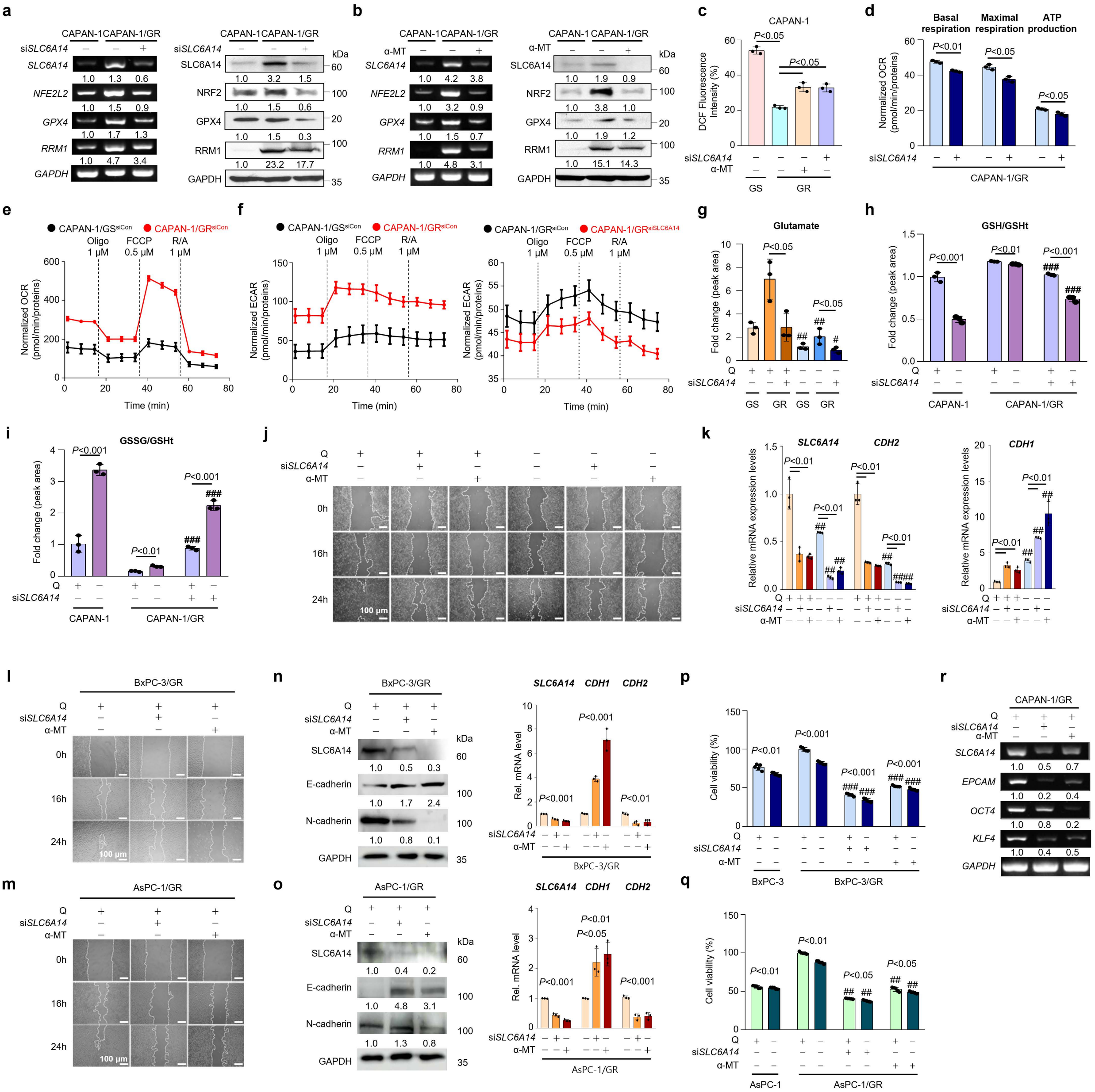

Supplementary Fig. 2. Increased expression of SLC6A14 leads to the utilization of glutamine, alleviating oxidative stress in gemcitabine-resistant PDAC.

a, **b** Antioxidant-related gene expression in CAPAN-1, CAPAN-1/GR, and CAPAN-1/GR cells transfected with siSLC6A14 (**a**) or treated with α-MT for 24 h (**b**), followed by qRT-PCR analysis (left) and western blotting analysis (right). **c** Relative ROS levels in CAPAN-1, CAPAN-1/GR siCon, CAPAN-1/GR with siSLC6A14 or with α-MT (α-methyl-DL-tryptophan) treatment for 24 h, measured by flow cytometry. **d** Basal/maximal respiration and ATP production in CAPAN-1/GR siCon versus

siSLC6A14. **e** OCR in CAPAN-1 and CAPAN-1/GR cells. **f** ECAR in CAPAN-1, CAPAN-1/GR siCon, and CAPAN-1/GR siSLC6A14. **g** The peak area of glutamate in CAPAN-1, CAPAN-1/GR siCon, and CAPAN-1/GR siSLC6A14 incubated for 24 h with a medium containing ± glutamine was measured by LC-MS analysis. **h**, **i** GSH/GSHt (**h**) and GSSG/GSHt (**i**) ratios in CAPAN-1, CAPAN-1/GR, and CAPAN-1/GR transfected with siSLC6A14, then incubated for 24 h with ± glutamine, followed by LC-MS analysis. **j** Wound-healing assay in CAPAN-1/GR cells with siSLC6A14 or α-MT for 24 h, then incubated with ± glutamine, quantified migration across the wound gap at indicated time points (40X magnification). **k** CAPAN-1/GR siCon, CAPAN-1/GR siSLC6A14 or α-MT for 24 h, then incubated with ± glutamine for 24 h, followed by qRT-PCR. **l**, **m** Wound-healing assay in BxPC-3/GR and AsPC-1/GR cells with siSLC6A14 or α-MT for 24 h. **n**, **o** EMT gene expression in BxPC-3/GR and AsPC-1/GR, BxPC-3/GR and AsPC-1/GR with siSLC6A14 or α-MT for 24 h, then incubated with ± glutamine, followed by western blotting (left) and qRT-PCR analysis (right). **p**, **q** WST assay in BxPC-3 and AsPC-1, BxPC-3/GR and AsPC-1/GR, BxPC-3/GR and AsPC-1/GR with siSLC6A14 or α-MT for 24 h, then incubated with ± glutamine for 24 h. **r** mRNA expression of stemness-related genes in CAPAN-1/GR, CAPAN-1/GR cells transfected with siSLC6A14, or treated with α-MT for 24 h was assessed. Scale bars, 100 μm (**j**, **l**, **m**). Error bars, mean ± SD, #$p < 0.05$; ##$p < 0.01$; ###$p < 0.001$; n.s., not significant; by one-way ANOVA (**c**, **k**, **n**, **o**) or Student's t test (**d**, **g**, **h**, **i**, **p**, **q**).

Supplementary Fig. 3

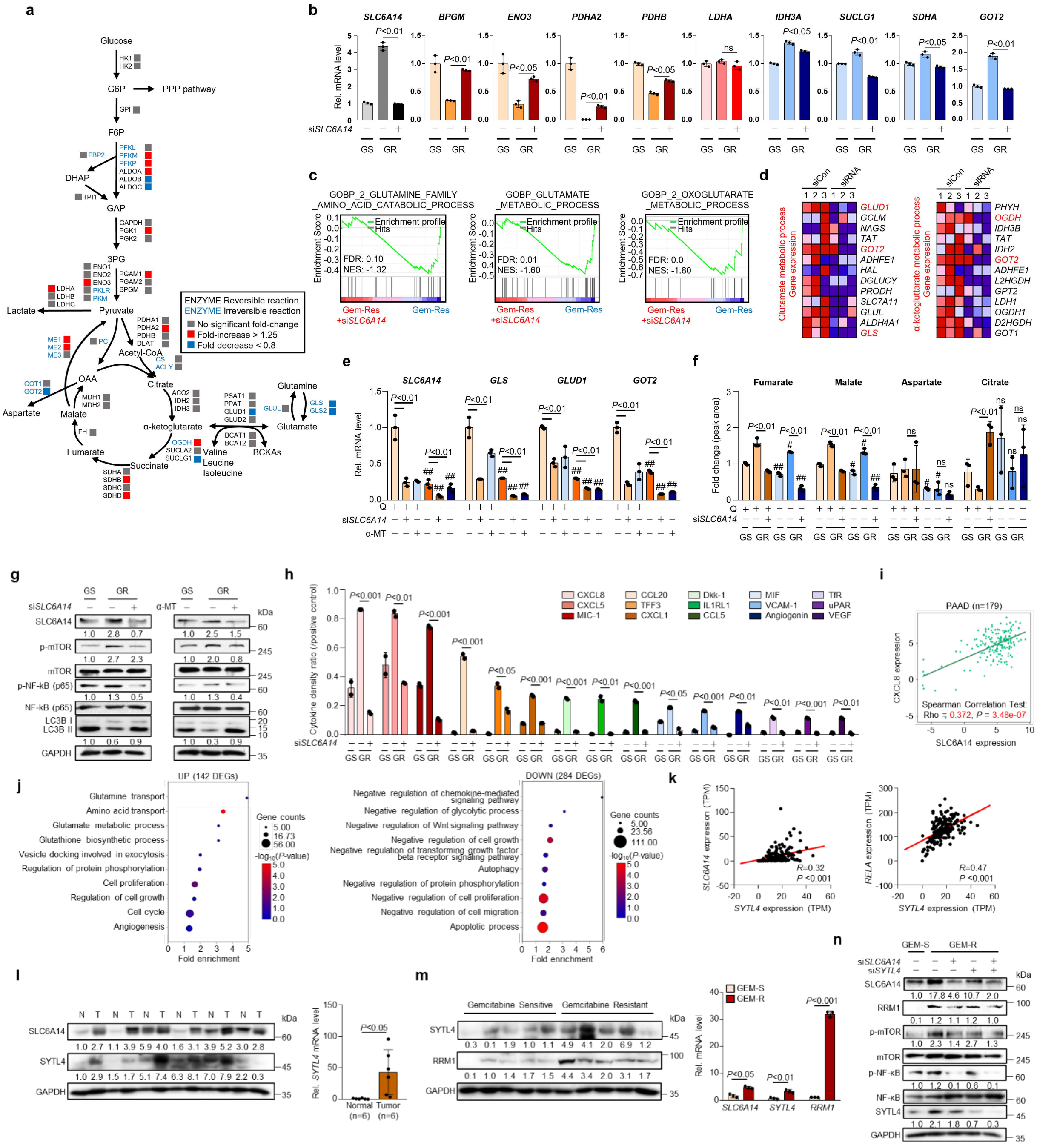

Supplementary Fig. 3. SLC6A14 increases α-KG levels in drug-resistant cancer than drug-sensitive cancer.

a Differential expression of genes encoding metabolic enzymes in CAPAN-1/GR transfected with siSLC6A14 relative to CAPAN-1/GR. **b** Expression levels of metabolic enzymes in SLC6A14, BPGM, ENO3, PDHA2, PDHB, LDHA, IDH3A, SUCLG1, SDHA, and GOT2 in CAPAN-1, CAPAN-1/GR, CAPAN-1/GR transfected with siSLC6A14 with ± glutamine, followed by qRT-PCR analysis. **c** Gene

Set Enrichment Analysis of glutamine family amino acid catabolism, glutamate metabolism, and 2-oxoglutarate metabolic processes in CAPAN-1/GR cells siCon versus si*SLC6A14*. **d** Heatmap showing the expression level of downregulated genes upon SLC6A14 knockdown in glutamate metabolic, and α-KG metabolic process. **e** Glutaminolysis-related enzyme genes in CAPAN-1/GR siCon, CAPAN-1/GR si*SLC6A14* or α-MT treatment for 24 h, then incubated with a medium containing ± glutamine for 24 h, followed by qRT-PCR analysis. **f** The peak area of fumarate, malate, aspartate, and citrate in CAPAN-1, CAPAN-1/GR, and CAPAN-1/GR transfected with siSLC6A14 incubated for 24 h with ± glutamine was measured via LC-MS analysis. **g** Expression levels of SLC6A14, p-mTOR, mTOR, p-NF-κB, NF-κB, LC3BI/II in CAPAN-1, CAPAN-1/GR, and CAPAN-1/GR either transfected with siSLC6A14 (left) or treated with α-MT for 24 h (right), then incubated for 24 h with ± glutamine. **h** Relative cytokine densitometry levels normalized from positive reference. **i** Correlation between SLC6A14 and CXCL8 expression in pancreatic cancer (n = 179). **j** DAVID-based gene ontology bubble plot analyses of upregulated and downregulated genes in CAPAN-1/GR cells compared to those in CAPAN-1 cells. **k** Correlation analyses of SYTL4 between either SLC6A14 or RELA in TCGA database. **l** Expression levels of SLC6A14 and SYTL4 in normal (n = 6) and tumor (n = 6) tissues, followed by western blot and qRT-PCR analysis. **m** SLC6A14, SYTL4, and RRM1 expression levels in gemcitabine-sensitive (n = 5) and gemcitabine-resistant (n = 5) tissues, followed by western blot and qRT-PCR analysis. **n** Western blot analysis of SLC6A14, p-mTOR, mTOR, p-NF-κB, and NF-κB in CAPAN-1, CAPAN-1/GR siCon, and CAPAN-1/GR siSLC6A14 incubated for 24 h with ± glutamine. Error bars, mean ± SD, #p < 0.05; ##p < 0.01; ###p < 0.001; n.s., not significant; by one-way ANOVA (**e**, **f**) or Spearman Correlation test (**i**) or Student's t test (**b**, **f**, **h**, **l**, **m**).

Supplementary Fig. 4

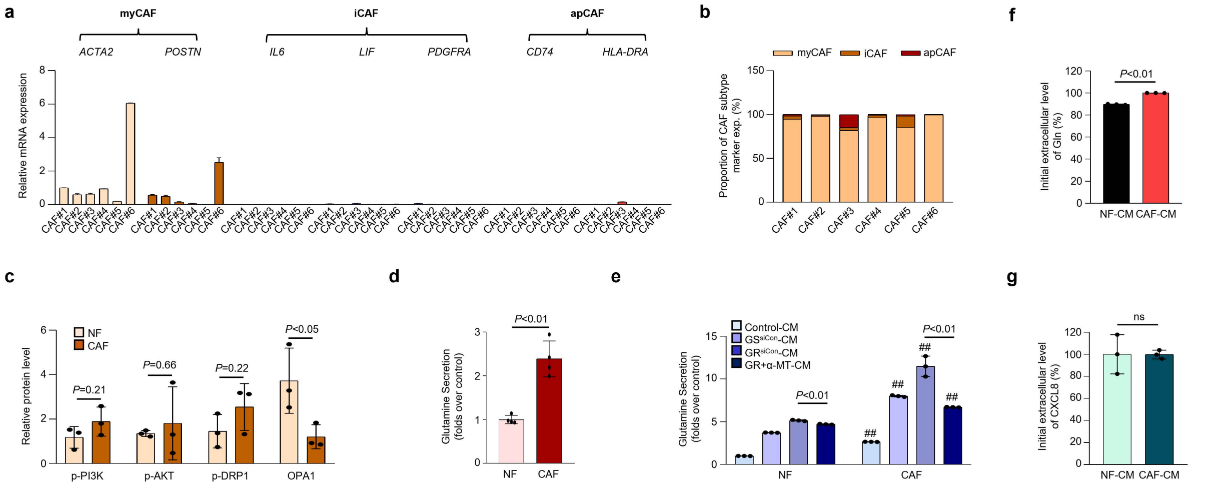

Supplementary Fig. 4. SLC6A14 is positively correlated with CXCL-8-inducing glutamine secretion from surrounding CAFs.

a Primary human CAFs (n = 6) were analzyed by qRT-PCR for markers of myCAF (*ACTA2*, *POSTN*), iCAF (*IL6*, *LIF*, *PDGFRA*), and apCAF (*CD74*, *HLA-DRA*) subtypes. **b** Proportion of CAF subtype marker expression. **c** Protein expression of p-PI3K/PI3K, p-DRP1/DRP1, and OPA1/GAPDH in NFs (n = 3) and CAFs (n = 3). **d** Glutamine secretion levels in NF and CAF. **e** Glutamine secretion level of NF and CAF followed by 24 h co-culture with control, CAPAN-1, CAPAN-1/GR, and CAPAN-1/ GR followed by 24 h treatment of α-MT-derived conditioned medium. **f** Initial secreted extracellular glutamine levels in NF- and CAF-conditioned media. **g** Initial extracellular CXCL8 levels in NF- and CAF-conditioned media. Error bars, mean ± SD, #p < 0.05; ##p < 0.01; ###p < 0.001; n.s., not significant; by Student's t test (**c**, **d**, **e**, **f**, **g**).

Supplementary Fig. 5

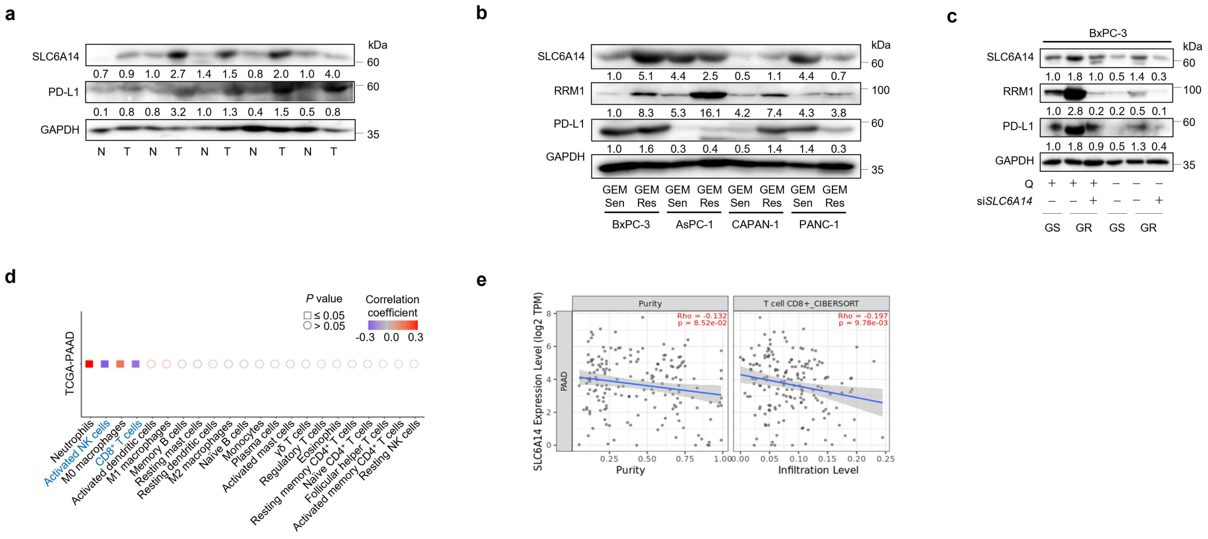

Supplementary Fig. 5. SLC6A14 attenuates T cell infiltration through PD-L1 regulation.

a SLC6A14, PD-L1 expression levels in normal pancreatic (n = 5) and PDAC tissue samples (n = 5), followed by western blotting. **b** Expression levels of SLC6A14, RRM1, and PD-L1 in AsPC-1, BxPC-3, CAPAN-1, and PANC-1 gemcitabine-sensitive and gemcitabine-resistant pancreatic cancer cell lines, followed by western blotting analysis. **c** Western blot analysis of SLC6A14, RRM1, and PD-L1 in BxPC-3, BxPC-3/GR siCon, and BxPC-3/GR si*SLC6A14* incubated for 24 h with ± glutamine. **d** Immune infiltration analysis was performed using database. **e** Correlation analysis of *SLC6A14* and $CD8^+$ T cell infiltration level was performed using TIMER 2.0 (Tumor IMmune Estimation Resource).

Supplementary Fig. 6

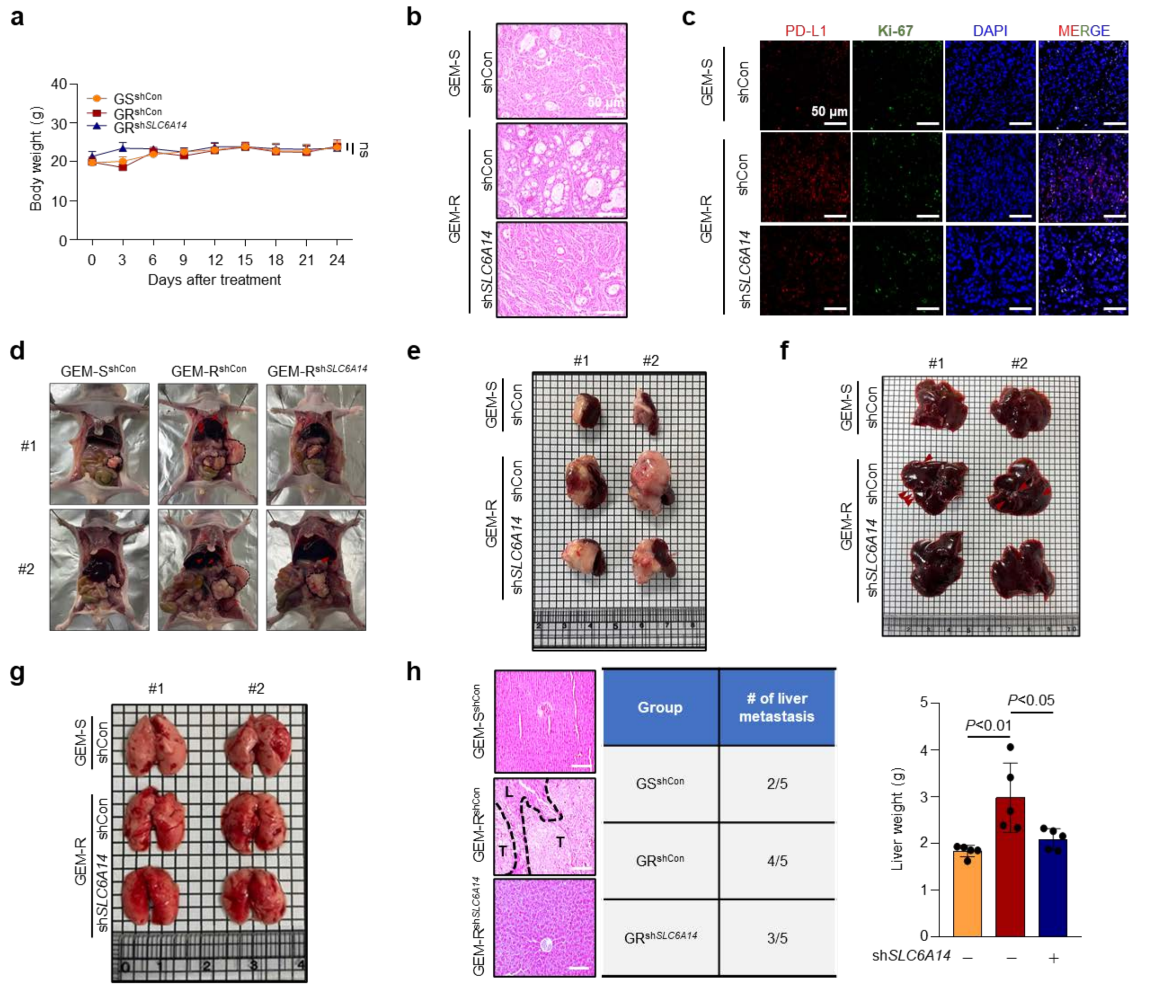

Supplementary Fig. 6. Blockade of SLC6A14 inhibits glutamine production suppressing tumorigenesis and liver metastasis.

a Body weight. **b** H&E staining of pancreatic tissues. **c** Immunofluorescence staining of PD-L1 (red), Ki-67 (green), and DAPI (blue) in the nuclei. (n = 5). **d** Representative images of anatomized mice. **e**–**g** Orthotopic mice pancreas (**e**), livers (**f**), and lungs (**g**). (**h**) H&E staining of liver (left) and number of liver metastasis (n = 5) (right). Scale bars, 50 μm (**b**, **c**, **h**). Error bars, mean ± SD, #p < 0.05; ##p < 0.01; ###p < 0.001; n.s., not significant; by one-way ANOVA (**a**, **h**).

Supplementary Fig. 7

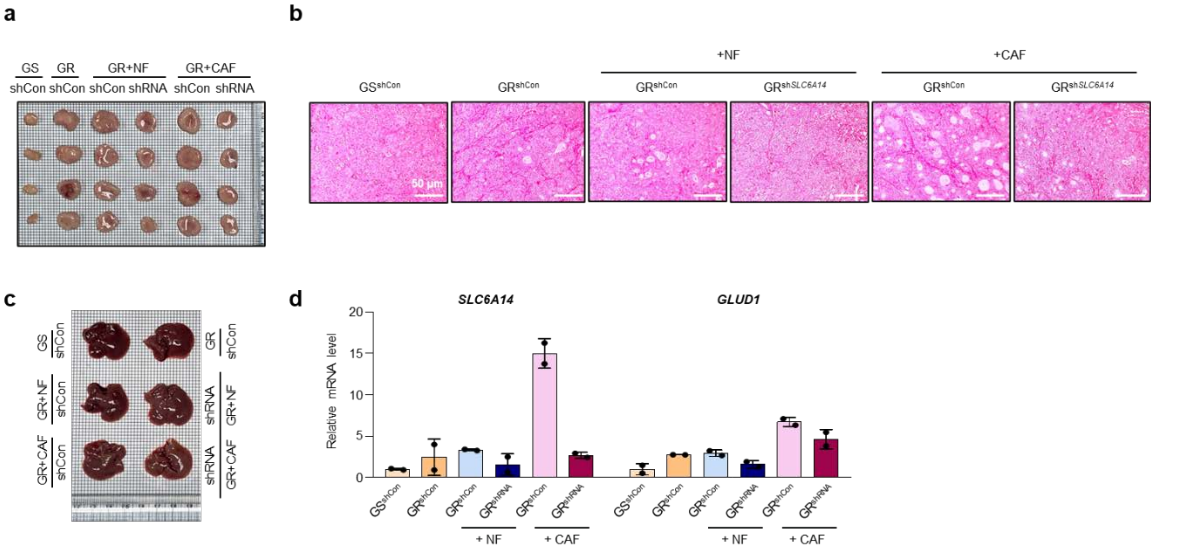

Supplementary Fig. 7. SLC6A14 knockdown attenuates fibroblast activation, but no liver metastasis.

a Representative image of pancreatic tumors (n = 4). **b**, **c** BALB/c nude mice were subcutaneously injected with CAPAN-1 or CAPAN-1/GR cells (2×10^5), with or without NFs or CAFs (3×10^5). After 3 weeks, lentivirus particles carrying shControl or shSLC6A14 were injected (2×10^6 particles) via the retro-orbital route. After 1 week, 10 mg/kg gemcitabine (n = 5) was administered intraperitoneally twice a week. (**b**) H&E staining. (**c**) Representative images of the liver tissue. (**d**) Expressions of SLC6A14 and GLUD1 in tumor tissues, followed by qRT-PCR analysis. Scale bars, 50 μm (**b**). Error bars, mean ± SD, #p < 0.05; ##p < 0.01; ###p < 0.001; n.s., not significant; by one-way ANOVA (**d**).

Supplementary Table 1. Reagents or resource, and primer sequences

Antibodies	Manufacturer	Catalog # and identifier
Rabbit polyclonal anti-SLC6A14	Thermo Fisher Scientific	Cat# PA5-87998, RRID: AB_2804576
Rabbit monoclonal anti-RRM1	Abcam	Cat# ab137114
Rabbit polyclonal anti-alpha smooth muscle actin	Abcam	Cat# ab5694, RRID: AB_2223021
Mouse monoclonal anti-granuphilin-A	Santa Cruz Biotechnology	Cat# sc-374544, RRID: AB_10988947
Mouse monoclonal anti-NRF2	Santa Cruz Biotechnology	Cat# sc-365949, RRID: AB_10917561
Mouse monoclonal anti-GPX4	Santa Cruz Biotechnology	Cat# sc-166570, RRID: AB_2112427
Mouse monoclonal anti-GAPDH	Santa Cruz Biotechnology	Cat# sc-365062, RRID: AB_10847862
Mouse monoclonal anti-OCT3/4	Santa Cruz Biotechnology	Cat# sc-5279, RRID: AB_628051
Mouse monoclonal anti-DRP1	Santa Cruz Biotechnology	Cat# sc-101270, RRID: AB_2093545
Mouse monoclonal anti-OPA1	Santa Cruz Biotechnology	Cat# sc-393296
Mouse monoclonal anti-E-cadherin	BD BioScience	Cat# 610181, RRID: AB_397580
Mouse monoclonal anti-N-cadherin	BD BioScience	Cat# 610920, RRID: AB_398236
Mouse monoclonal anti-EpCAM	Cell Signaling Technology	Cat# 2929S, RRID: AB_2098657
Rabbit monoclonal anti-KLF4	Cell Signaling Technology	Cat# 12173S, RRID: AB_2797840
Rabbit polyclonal anti-phospho-mTOR (Ser2481)	Cell Signaling Technology	Cat# 2974S, RRID: AB_2262884
Rabbit monoclonal anti-mTOR	Cell Signaling Technology	Cat# 2983S, RRID: AB_2105622
Rabbit monoclonal anti-p-NF-κB (Ser536)	Cell Signaling Technology	Cat# 3033S, RRID: AB_331284
Rabbit monoclonal anti-NF-κB	Cell Signaling Technology	Cat# 8242S, RRID: AB_10859369
Rabbit polyclonal anti-LC3B	Cell Signaling Technology	Cat# 2775S, RRID: AB_915950
Rabbit polyclonal anti-p-DRP1 (Ser616)	Cell Signaling Technology	Cat# 3455S, RRID: AB_2085352
Rabbit polyclonal anti-Ki67	Abcam	Cat# ab15580, RRID: AB_443209
HRP-conjugated goat anti-mouse secondary	Cell Signaling Technology	Cat# 7076S, RRID: AB_330924
HRP-conjugated goat anti-rabbit secondary	Cell Signaling Technology	Cat# 7074S, RRID: AB_2099233
Goat anti-Rabbit IgG (H+L) Cross-adsorbed secondary antibody, Texas Red-X	Invitrogen	Cat# T-6391, RRID: AB_2556779
Goat anti-Mouse (IgG (H+L) Cross-adsorbed secondary antibody, Alexa Fluor™ 488	Invitrogen	Cat# A-11001, RRID: AB_2534069
Human monoclonal anti-CD3 (OKT3)	eBioscience™	Cat# 16-0037-81, RRID: AB_468854
Human monoclonal anti-CD28 (CD28.2)	eBioscience™	Cat# 16-0289-81, RRID: AB_468926

**Virus strains**	**Manufacturer**	**Catalog # and identifier**
Human SLC6A14 shRNA lentiviral particles	OriGene Technology	Cat# TL309295V
**Biological samples**	**Manufacturer**	**Catalog # and identifier**
Human PDAC samples	Gangnam Severance Hospital	N/A
**Chemicals, peptides, and recombinant proteins**	**Manufacturer**	**Catalog # and identifier**
TRIzol	Thermo Fisher Scientific	Cat# 15596-018
DMEM, high glucose	Biowest	Cat# L0103-500
DMEM-F12	Biowest	Cat# L0092-500
RPMI 1640, high glucose	Biowest	Cat# L0498-500
RPMI 1640 w/o L-glutamine	Biowest	Cat# L0501-500
1x DPBS	Biowest	Cat# L0615-500
Fetal bovine serum	Biowest	Cat# S1480-500
Trypsin-EDTA (0.25%)	Gibco	Cat# 25200-072
Bovine serum albumin	BOVOGEN	Cat# BSAS0.1
Antibiotic-antimycotic	Gibco	Cat# 15240-062
Lipofectamine^{TM} RNAiMAX Transfection Reagent	Thermo Fisher Scientific	Cat# 13778075
Collagenase P	Sigma-Aldrich	Cat# 11213865001
Hank's Balanced Salt Solution	Biowest	Cat# L0607-500
Opti-MEM	Gibco	Cat# 31985-070
Skim milk	DIFCO	Cat# 232100
α-Methyl-DL-tryptophan	Sigma-Aldrich	Cat# M8377
Gemcitabine	YUHAN	Cat# L01BC05
Ez-Cytox	DoGENBio	Cat# EZ-3000
FluoroShield mounting medium with DAPI	Abcam	Cat# ab104139
10x Tris-Buffered Saline, 0.1% Tween-20	Cellnest	Cat# CNT007-1000
Goat serum	Thermo Fisher Scientific	Cat# 16210064
Crystal violet	JUNSEI	Cat# C1065-25g
**Critical commercial assays**	**Manufacturer**	**Catalog # and identifier**
Glutamine Assay Kit	Abcam	Cat# ab197011
Human IL-8/CXCL8 Quantikine ELISA Kit	R&D SYSTEM	Cat# D8000C
Human IFN-γ Quantikine ELISA Kit	R&D SYSTEM	Cat# DIF50C
Human Pan T Cell Isolation Kit	Miltenyi Biotec	Cat# 130-096-535
Human XL Cytokine Array Kit	R&D SYSTEM	Cat# ARY022B

Power SYBR Green PCR Master Mix	ABI	Cat# 4368706
Maxime PCR PreMix (i-StarTaq)	IntronBio	Cat# 25167
Maxime RT-PCR PreMix	IntronBio	Cat# 25131
Maxime RT PreMix	IntronBio	Cat# 25081
Clarity Western ECL Substrate	Bio-Rad	Cat# 1705061
SuperSignal™ West Atto Ultimate Sensitivity Substrate	Thermo Fisher Scientific	Cat# A38555
6.5 mm Transwell® with 8.0 µm pore PET membrane	Corning	Cat# 3464
Bio-Rad protein assay kit	Bio-Rad	Cat# 5000006
**Deposited data**	**Manufacturer**	**Catalog # and identifier**
Raw and processed sequencing data	Steele et al., Werba et al., Perwez et al., Liss et al.	GEO: GSE155698, GSE212966, GSE183795, GSE164665
Supplementary Data 2. Unprocessed source data underlying all blots and graphs	This paper	N/A
**Cell lines**	**Manufacturer**	**Catalog # and identifier**
Human AsPC-1 pancreatic adenocarcinoma	ATCC	Cat# CRL-1682, RRID: CVCL_0152
Human BxPC-3 pancreatic adenocarcinoma	ATCC	Cat# CRL-1687, RRID: CVCL_0186
Human CAPAN-1 pancreatic adenocarcinoma	ATCC	Cat# HTB-79, RRID: CVCL_0237
Human MIA PaCa-2 pancreatic adenocarcinoma	ATCC	Cat# CRL-1420, RRID: CVCL_0428
Human PANC-1 pancreatic adenocarcinoma	ATCC	Cat# CRL-1469, RRID: CVCL_0480
**Organisms**	**Manufacturer**	**Catalog # and identifier**
BALB/c nude	Model Animal Research Center of Yonsei University	N/A
**Primer sequences**	**Manufacturer**	**Catalog # and identifier**
Human SLC6A14 primers: Forward: ATCGTCTGGCAAGGTGGTAT; Reverse: TGAGTGGCAGCATCTTTCCAT	Macrogen, Inc.	N/A
Human RRM1 primers: Forward: TCTCAGACGGAAACAGGCAC; Reverse: GCACAGGTTGCTGCATTTGA	Macrogen, Inc.	N/A
Human GAPDH primers: Forward: GTCTCCTCTGACTTCAACAGCG; Reverse: ACCACCCTGTTGCTGTAGCCAA	Macrogen, Inc.	N/A

Human NFE2L2 primers: Forward: TCCAGTCAGAAACCAGTGGAT; Reverse: GAATGTCTGCGCCAAAAGCTG	Macrogen, Inc.	N/A
Human GPX4 primers: Forward: AGAGATCAAAGAGTTCGCCGC; Reverse: TCTTCATCCACTTCCACAGCG	Macrogen, Inc.	N/A
Human CDH1 primers: Forward: GCTCCTGAAAAGAGAGTGGAAG; Reverse: TGGCAGTGTCTCTCCAAATCCG	Macrogen, Inc.	N/A
Human CDH2 primers: Forward: CCTCCAGAGTTTACTGCCATGAC; Reverse: GTAGGATCTCCCCCACTGATTC	Macrogen, Inc.	N/A
Human EPCAM primers: Forward: TGCTGGAATTGTTGTGCTGG; Reverse: AAGATGTCTTCGTCCCACGC	Cosmo Genetech Co. Ltd.	N/A
Human OCT4 primers: Forward: CTCACCCTGGGGGTTCTATT; Reverse: CTGGTTCGCTTTCTCTTTCG	Cosmo Genetech Co. Ltd.	N/A
Human KLF4 primers: Forward: ACACACGGGATGATGCTCAC; Reverse: CGCGTAATCACAAGTGTGGG	Macrogen, Inc.	N/A
Human MYC primers: Forward: GGTAGTGGAAAACCAGCAGCC; Reverse: TTCTCCTCCTCGTCGCAGTA	Macrogen, Inc.	N/A
Human GLS1 primers: Forward: TCTACAGGATTGCGAACGTCT; Reverse: CTTTGTCTAGCATGACACCATCT	Macrogen, Inc.	N/A
Human GLUD1 primers: Forward: GGGCTTTATTGGTCCTGGCA; Reverse: AGATGCGTCCATGGATTCCC	Macrogen, Inc.	N/A
Human GOT2 primers: Forward: TTGAAGAGTGGCCGGTTTGT; Reverse: GGCAGAAAGACATCTCGGCT	Macrogen, Inc.	N/A
Human BPGM primers: Forward: CTCGGAACTGTGGGAAGCAA; Reverse: CGCTCATTTAGACGCCAGGA	Macrogen, Inc.	N/A
Human ENO3 primers: Forward:	Macrogen, Inc.	N/A

TTTGCCCGGGAAATCTTGGA; Reverse: GCCTTCAGGACTCCTTTCCC		
Human PDHA2 primers: Forward: GTGGCATCCCGTAACTCCTC; Reverse: TTCAATTCCATGCGGCGAAC	Macrogen, Inc.	N/A
Human PDHB primers: Forward: AAGAGGCGCTTTCACTGGA; Reverse: CACAGCCCTCGACTAACCTT	Macrogen, Inc.	N/A
Human LDHA primers: Forward: TGTCTCTGGCAAAGTGGATATCTT; Reverse: ATCCAGATTGCAACCGCTTC	Macrogen, Inc.	N/A
Human IDH3A primers: Forward: CGCGTGGATCTCTAAGGTCTC; Reverse: GGTGCTTTGGCAGCATCAAA	Macrogen, Inc.	N/A
Human SUCLG1 primers: Forward: CAACTGCCCTGGAGTCATCA; Reverse: CCAATGCCAACGCACAAAGA	Macrogen, Inc.	N/A
Human SDHA primers: Forward: GCATGCCAGGGAAGACTACA; Reverse: AGTTTTGTCGATCACGGGTCT	Macrogen, Inc.	N/A
Human CXCR2 primers: Forward: CTCAACCCCCTCATCTACGC; Reverse: GCCCTGAAGAAGAGCCAACA	Macrogen, Inc.	N/A
Human ACTA2 primers: Forward: ACTGCCTTGGTGTGTGACAA; Reverse: CACCATCACCCCCTGATGTC	Macrogen, Inc.	N/A
Human DNM1L primers: Forward: TCACCCGGAGACCTCTCATT; Reverse: TCTGCTTCCACCCCATTTTCT	Macrogen, Inc.	N/A
Human FIS1 primers: Forward: GTAAAGGCATCGTGCTGCTC; Reverse: CTCGTATTCCTTGAGCCGGT	Macrogen, Inc.	N/A
Human MFF primers: Forward: CCACCTCGTGTACTTACGCT; Reverse: TGCCAACTGCTCGGATTTCT	Macrogen, Inc.	N/A
Human OPA1 primers: Forward: TGGAATGACTTTGCGGAGGA; Reverse:	Macrogen, Inc.	N/A

GCTGCATCCCATTGCTGTTT		
Human MFN1 primers: Forward: AATGCTCAAAGGGTGCTCCT; Reverse: GCATTATCTGGCGTTGCTGG	Macrogen, Inc.	N/A
Human MFN2 primers: Forward: GTCTGACCTGGACCACCAAG; Reverse: TGCAGTTGGAGCCAGTGTAG	Macrogen, Inc.	N/A
Human SYTL4 primers: Forward: TGGCACTGGGATCAGTAATGG; Reverse: CTTGGCCATTGAGGAACGGA	Macrogen, Inc.	N/A
Human POSTN primers: Forward: CATTGATGGAGTGCCTGTGG; Reverse: GTGACCTTGGTGACCTCTTCT	Macrogen, Inc.	N/A
Human IL6 primers: Forward: ACTCCTTCTCCACAATACCCC; Reverse: CAGTGCCTCTTTGCTGCTTTC	Macrogen, Inc.	N/A
Human LIF primers: Forward: CTTGGCGGCAGGAGTTGT; Reverse: GTTGTTGTGACATGGGTGGC	Macrogen, Inc.	N/A
Human PDGFRA primers: Forward: CGACAGCAGACAGGGCTTTA; Reverse: AACAGCACAGGTGACCACAA	Macrogen, Inc.	N/A
Human CD74 primers: Forward: GCACTCCTTGGAGCAAAAGC; Reverse: AAGACACACCAGCAGTAGCC	Macrogen, Inc.	N/A
Human HLA-DRA primers: Forward: GAGAGCCCAACGTCCTCATC; Reverse: GGAACTTGCGGAAAAGGTGG	Macrogen, Inc.	N/A
Human SLC2A1 primers: Forward: TGGCATCAACGCTGTCTTCT; Reverse: AACAGCGACACGACAGTGAA	Macrogen, Inc.	N/A
Human SLC16A1 primers: Forward: TTTCTTTGCGGCTTCCGTTG; Reverse: CTCTGGGGTCCAACAAGGTC	Macrogen, Inc.	N/A
Human SLCO4A1 primers: Forward: AAGGCCACCTGAACCTAACG; Reverse: GTCTCGGTACACCTTCTGGC	Macrogen, Inc.	N/A

Human SLC35F2 primers: Forward: GCGCAGGATAAAAGGCAAACT; Reverse: GAAGCATGGGGGTGTTCACT	Macrogen, Inc.	N/A
Human SLC9A2 primers: Forward: GCTATTTCATGCCCACTCGC; Reverse: GCAAAGTGATGTCGCTGAGG	Macrogen, Inc.	N/A
Human HK1 primers: Forward: ATGGCTGCGGTTGTGGATAA; Reverse: TCACCGTCTGGTGCATGATT	Macrogen, Inc.	N/A
Human HK2 primers: Forward: TCCCCTGCCACCAGACTAAA; Reverse: GGATCAGAGCCACAACGTCT	Macrogen, Inc.	N/A
Human GPI primers: Forward: AATCTGGAACCCGTGTGGAC; Reverse: GACCGGGATGAGGAAGTCAC	Macrogen, Inc.	N/A
**Software and algorithms**	**Manufacturer**	**Catalog # and identifier**
Imagej	https://imagej.nih.gov/ij/	https://imagej-nihgove.gate2.inist.fr/ij/
GraphPad Prism 8.0	GraphPad Software	https://www.graphpad.com/features
FlowJo	BD Bioscience	N/A
Rstudio	Rstudio Team, 2023	http://www.rstudio.com
GSEA	Broad Institute	https://www.gsea-msigdb.org/gsea/ index.jsp
Seurat package 4.0.5	Stuart et al.	https://satijalab.org/seurat/
